# Ecological Dichotomies Arise in Microbial Communities Due to Mixing of Deep Hydrothermal Waters and Atmospheric Gas in a Circumneutral Hot Spring

**DOI:** 10.1128/AEM.01598-21

**Published:** 2021-11-10

**Authors:** Maria C. Fernandes-Martins, Lisa M. Keller, Mason Munro-Ehrlich, Kathryn R. Zimlich, Madelyn K. Mettler, Alexis M. England, Rita Clare, Kevin Surya, Everett L. Shock, Daniel R. Colman, Eric S. Boyd

**Affiliations:** a Department of Microbiology and Cell Biology, Montana State University, Bozeman, Montana, USA; b Department of Chemical and Biological Engineering, Montana State University, Bozeman, Montana, USA; c Department of Chemistry and Biochemistry, Montana State University, Bozeman, Montana, USA; d School of Molecular Sciences, Arizona State University, Tempe, Arizona, USA; e School of Earth and Space Exploration, Arizona State University, Tempe, Arizona, USA; University of Michigan-Ann Arbor

**Keywords:** aerobic, anaerobic, autotrophic, heterotrophic, oxygen availability, dissolved organic carbon, archaea, circumneutral hot spring, silica, subsurface, Yellowstone

## Abstract

Little is known of how the confluence of subsurface and surface processes influences the assembly and habitability of hydrothermal ecosystems. To address this knowledge gap, the geochemical and microbial composition of a high-temperature, circumneutral hot spring in Yellowstone National Park was examined to identify the sources of solutes and their effect on the ecology of microbial inhabitants. Metagenomic analysis showed that populations comprising planktonic and sediment communities are archaeal dominated, are dependent on chemical energy (chemosynthetic), share little overlap in their taxonomic composition, and are differentiated by their inferred use of/tolerance to oxygen and mode of carbon metabolism. The planktonic community is dominated by putative aerobic/aerotolerant autotrophs, while the taxonomic composition of the sediment community is more evenly distributed and comprised of anaerobic heterotrophs. These observations are interpreted to reflect sourcing of the spring by anoxic, organic carbon-limited subsurface hydrothermal fluids and ingassing of atmospheric oxygen that selects for aerobic/aerotolerant organisms that have autotrophic capabilities in the water column. Autotrophy and consumption of oxygen by the planktonic community may influence the assembly of the anaerobic and heterotrophic sediment community. Support for this inference comes from higher estimated rates of genome replication in planktonic populations than sediment populations, indicating faster growth in planktonic populations. Collectively, these observations provide new insight into how mixing of subsurface waters and atmospheric oxygen create dichotomy in the ecology of hot spring communities and suggest that planktonic and sediment communities may have been less differentiated taxonomically and functionally prior to the rise of oxygen at ∼2.4 billion years ago (Gya).

**IMPORTANCE** Understanding the source and availability of energy capable of supporting life in hydrothermal environments is central to predicting the ecology of microbial life on early Earth when volcanic activity was more widespread. Little is known of the substrates supporting microbial life in circumneutral to alkaline springs, despite their relevance to early Earth habitats. Using metagenomic and informatics approaches, water column and sediment habitats in a representative circumneutral hot spring in Yellowstone were shown to be dichotomous, with the former largely hosting aerobic/aerotolerant autotrophs and the latter primarily hosting anaerobic heterotrophs. This dichotomy is attributed to influx of atmospheric oxygen into anoxic deep hydrothermal spring waters. These results indicate that the ecology of microorganisms in circumneutral alkaline springs sourced by deep hydrothermal fluids was different prior to the rise of atmospheric oxygen ∼2.4 Gya, with planktonic and sediment communities likely to be less differentiated than contemporary circumneutral hot springs.

## INTRODUCTION

Yellowstone National Park (YNP), Wyoming, hosts the world’s largest active continental volcanic, hydrothermal system (1), and its surficial expression forms the >10,000 hot springs, mudpots, fumaroles, and geysers distributed across the landscape (2). Each of these hydrothermal features has a unique geochemical composition (3) that supports unique and biodiverse microbial communities (4, 5). This is particularly true for hot springs with geochemical conditions that preclude photosynthesis, such as those with temperatures >73°C (6, 7). In such hot springs, microbial populations are dependent on chemical energy that is generated by dissipating disequilibrium in available electron donors and acceptors through redox-based metabolism (3, 8). As such, the availability of, and disequilibrium in, electron donors/acceptors and other nutrients exerts fundamental control on the taxonomic and functional diversity of high-temperature (>73°C) hot spring populations and their communities.

While high-temperature acidic hot springs tend to be dominated by aerobes that are supported by dissimilatory iron- and sulfur-based metabolisms (5, 9), far less is known of the energy sources that support microbial metabolism in circumneutral to alkaline hot springs. To assess energy sources that may support microbial metabolism in circumneutral to alkaline springs, metagenomic data from sediments or biofilms reported from 18 circumneutral to alkaline hot springs (pH ≥ 6.5) in YNP were analyzed for the abundance of proteins involved in a variety of energy metabolism functionalities (5). Sediment- or biofilm-associated communities from circumneutral to alkaline springs were found to be enriched in functionalities involving respiration of inorganic nitrogen compounds or oxygen (5). Perhaps consistent with this, a predominant bacterium identified in circumneutral to alkaline springs is *Thermocrinis* (10, 11), a genus comprising microaerophilic organisms (12). However, other studies have shown that a predominant archaeal genus commonly identified in waters and/or sediments of circumneutral to alkaline springs is *Pyrobaculum* (13). Some *Pyrobaculum* strains respire oxygen (14–16), whereas other strains are anaerobes that can respire ferric iron (17) or, in the case of Pyrobaculum yellowstonensis, compounds such as elemental sulfur or arsenate (18). The prevalence of P. yellowstonensis strains putatively capable of respiring arsenic in circumneutral to alkaline high-temperature springs in YNP (13) is consistent with elevated concentrations of arsenic, including arsenate, in this spring type (19).

Previous 16S rRNA gene studies have shown that communities inhabiting the water column (i.e., planktonic) and the sediments of hot springs can be differentiated by habitat type, both in YNP (13) and in other continental hot springs such as those in the Great Basin, United States (20), and Tengchong, China (21, 22). Colman et al. (13) show that this is particularly true for hot springs in YNP with circumneutral to alkaline pH that are sourced from the parent hydrothermal aquifer. Sediments in these springs often lack detectable redox active minerals (13), leading to the hypothesis that the electron donors and acceptors fueling microbial communities are in a soluble or dissolved form supplied by the volcanic system or by exogenous input. However, this hypothesis has yet to be robustly evaluated. An important consideration in evaluating such a hypothesis is that deep fluids sourced from the parent hydrothermal aquifer tend to be saturated with dissolved silica that can precipitate as sinter during their ascent and cooling as they migrate to the surface (23, 24). This is thought to seal the plumbing system feeding these springs, thereby limiting the input of dissolved organic carbon (DOC) and oxygen (O_2_) enriched near-surface groundwaters (25) and further constraining the spectrum of nutrients and energy sources available to microorganisms inhabiting these springs. Evidence in support of this comes from the lack of detection of tritium (half-life, 12.3 years) in hot spring waters sourced by the parent aquifer (26–28); tritium would be expected to be present in hot spring waters if recent meteoric water was input into these springs. Given the length of time that these waters have been out of contact with the atmosphere (>200 years [26]), it is likely that the waters discharging in this spring type are anoxic and lacking in electron acceptors for microbial metabolism other than SO_4_^2−^ and bicarbonate from parent hydrothermal fluid (2). The high temperatures of these discharging waters likely also limit extensive infusion of atmospheric O_2_, given the inverse relationship between oxygen solubility and temperature (8). Further, the high temperatures (>73°C) of these springs do not permit photosynthesis (6, 7) and, thus, endogenous production of photosynthetic DOC or O_2_. Thus, it is possible that previously observed habitat differentiation in planktonic and sediment communities inhabiting circumneutral to alkaline hot springs in YNP (13) and elsewhere (20–22) derives from differential availability of oxidants, in particular, atmospheric O_2_, given the limited availability of alternative oxidants in these environments.

In the present study, we hypothesized that hot springs sourced by the deep hydrothermal aquifer in YNP would host distinct sediment and planktonic microbial communities that are adapted to take advantage of differentially available electron donor/acceptor pairs in their respective habitats. Specifically, we hypothesized that populations comprising the planktonic community would be primarily aerobic, given the potential infusion of O_2_ through atmospheric ingassing, whereas populations inhabiting sediments would be primarily anaerobic due to limited availability of dissolved O_2_ in that habitat and consumption of O_2_ in the overlying water column. Further, we hypothesized that the populations comprising both planktonic and sediment communities would be primarily autotrophic, given the low concentrations of DOC present in fluids sourcing this spring type (25). We assessed these interrelated hypotheses through analyses of the geochemistry of hot spring waters and metagenomic sequencing of sediment and planktonic microbial communities. Metagenomic sequences were binned into population-level metagenome-assembled genomes (MAGs) and subjected to analyses of community structure, inferred metabolic capabilities, and estimated genome replication rates. The results are discussed in relation to the consequences of influx of atmospheric gas (i.e., O_2_) into reduced waters that are DOC limited on the ecology of planktonic and sediment communities inhabiting circumneutral to alkaline hot springs in YNP. The results provide insight into how the habitability and ecology of these early Earth analog environments may have changed as O_2_ began to accumulate in the atmosphere ∼2.4 billion years ago (Gya) (as reviewed in reference 29).

## RESULTS AND DISCUSSION

### Hot spring description.

**FIG 1:**
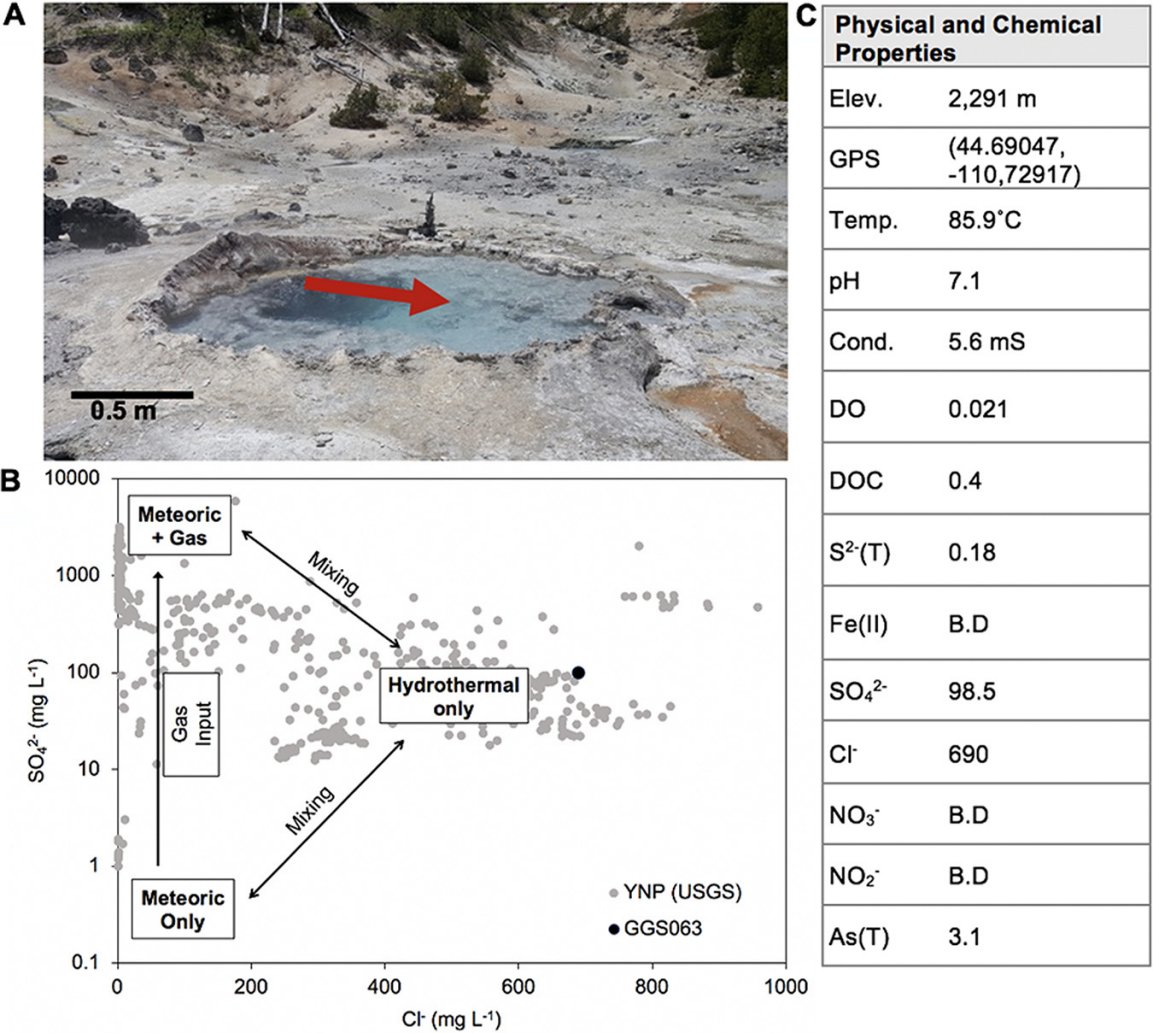
Physical and chemical properties of Gibbon Geyser Spring 063 (GGS063), Yellowstone National Park (YNP). (A) Image of GGS063, looking northwest, with the red arrow denoting the location within the spring where planktonic and sediment biomass was collected. (B) Plot of sulfate (SO_4_^2−^) and chloride (Cl^−^) concentrations in YNP springs sampled between the years 2003 and 2013 from U.S. Geological Survey (USGS) publications in gray (32, 90) and end member fluid compositions, based on a published model (29), denoted in black boxes. The measured concentrations for GGS063 are indicated by the black data point, which represents the sulfate and chloride values measured for GGS063. (C) Physical and select chemical measurements made for GGS063. All measured chemical data are available in Table S5 in the supplemental material. Elev., elevation; Temp., temperature; Cond., conductivity.; DO, dissolved oxygen; T, total; B.D, below detection. All chemical data are reported in mg liter^−1^.

Gibbon Geyser Spring 63 (National Park Service identifier GGS063) is a large (∼2 m diameter) (Fig. 1A), shallow (∼0.5 m depth), turbulent, and circumneutral hot spring (pH 7.1; temperature, 85.9°C) (Fig. 1C) located in the northern section of the Geyser Creek area of the Gibbon Geyser Basin in YNP. Concentrations of sulfate (98.6 mg liter^−1^) and chloride (690 mg liter^−1^) are consistent with this spring being sourced by the parent hydrothermal aquifer (Fig. 1B) (2, 29). Thus, the spring is representative of >50% of hot springs in YNP that are also thought to be sourced primarily by this aquifer (9). Like many high-temperature circumneutral to alkaline springs, the sulfate and chloride in GGS063 are likely further concentrated due to boiling and evaporation (29). As such, dissolved solutes in the spring, which are delivered from the aquifer sourcing the spring, are presumed to be uniform in the pool. Concentrations of nitrate and nitrite were below the limit of detection (Fig. 1C). This is also consistent with GGS063 being sourced by the parent hydrothermal aquifer with limited input of surface waters since nitrate is a common solute in hot springs impacted by input of near-surface groundwaters (30). The concentration of DOC was low (0.4 mg liter^−1^) and is near the detection limit of the method used. The low concentration of DOC in GGS063 is consistent with low DOC concentrations measured in springs in YNP (and Tengchong, China) sourced by the parent hydrothermal aquifer (25), as discussed in more detail below.

GGS063 had a low concentration of dissolved O_2_ (0.021 mg liter^−1^ or 1.3 μM) that was near the limit of detection of the probe used (0.015 mg liter^−1^ or 0.9 μM). Dissolved O_2_ was measured at a depth of ∼20 cm beneath the air-water interface, and it is possible that spatial variability exists in the measurement, given the turbulence of the spring. While spatial variation of dissolved O_2_ (or other dissolved components) was not assessed in this study, the low concentration of dissolved O_2_ measured in GGS063 is consistent with other circumneutral to alkaline hot springs in YNP (4) where temperatures are too high (>73°C) to support endogenous production of O_2_ through oxygenic photosynthesis (6, 7). Rather, the dissolved O_2_ measured in springs like GGS063 is due to atmospheric influx, which is limited at high temperatures (31).

The concentration of total arsenic (3.1 mg liter^−1^ or 41 μM) was high in GGS063 waters (Fig. 1) and is among the highest concentration of total arsenic measured in hydrothermal waters in YNP (19, 32; https://www.usgs.gov/mission-areas/water-resources/science/water-chemistry-data-selected-springs-geysers-and-streams?qt-science_center_objects=0#qt-science_center_objects). This is consistent with the high arsenic concentrations measured in hot springs sourced by the parent hydrothermal aquifer since the rhyolitic bedrock that hosts this aquifer is thought to be the source of As in these springs (19). While the oxidation state of arsenic measured in GGS063 was not determined in the present study, the amount of total arsenic as arsenite [As(III)] in other springs with similar chemical compositions tends to be >50%, with the balance presumed to be arsenate [As(V)] (19, 32; https://www.usgs.gov/mission-areas/water-resources/science/water-chemistry-data-selected-springs-geysers-and-streams?qt-science_center_objects=0#qt-science_center_objects). As(III) is the predominant form of arsenic in hot springs sourced by the parent hydrothermal aquifer (33) since its oxidation is slow in the absence of O_2_ and aerobic microorganisms (34). As such, As(III) is likely to be an available electron donor in GGS063, and As(V), generated as a product of aerobic microbial As(III) oxidation, may be an available electron acceptor. Other soluble inorganic electron donors that were measured include sulfide (0.18 mg liter^−1^ or 5.3 μM). Other soluble electron acceptors, such as ferric iron, were below the limit of detection. Although evidence for iron oxides or elemental sulfur were not visually apparent in GGS063 (Fig. 1A) and these minerals are not typical for springs with circumneutral to alkaline pH sourced by the parent hydrothermal aquifer (13, 35), their presence and potential use as electron acceptor and/or donor by sediment communities cannot be excluded. The complete list of measured aqueous chemical data is available in Table S5 in the supplemental material.

### Planktonic and sediment community composition and structure.

While the focus of the manuscript is a functional analysis of planktonic and sediment communities using metagenomics approaches, it was first necessary to ensure that the taxonomic composition of MAGs obtained from our metagenomes was reflective of the community 16S rRNA genes that were assembled from the metagenomes, as well as 16S rRNA genes from other high-temperature circumneutral to alkaline springs in YNP. Indeed, the taxonomic composition of MAGs and 16S rRNA genes extracted from metagenomic assemblies was similar in both the planktonic and sediment communities (Fig. S1A). Further, comparison of the composition of 16S rRNA genes obtained from planktonic and sediment communities from GGS063 to those compiled from the water column and sediments from other circumneutral to alkaline hot spring communities in YNP (13) shows that they are also highly similar, with the exception of *Desulfurococcales*, which were particularly abundant in the sediment metagenome but largely absent in the previously reported PCR-amplified 16S rRNA gene libraries (Fig. S1A and B). The recovery of *Desulfurococcales* MAGs and 16S rRNA genes in GGS063 versus 16S rRNA gene amplicons from other hot springs is likely attributable to the primers (515F-806R [36]) that were used to amplify 16S rRNA genes in the previous study that are now known to be biased against the amplification of key groups of *Archaea* (37), including members of the *Desulfurococcales* (38). Collectively, these data indicate that the taxonomic and functional insights generated from MAGs from planktonic and sediment communities in GGS063 are reflective of other communities inhabiting circumneutral to alkaline springs in YNP.

**FIG 2:**
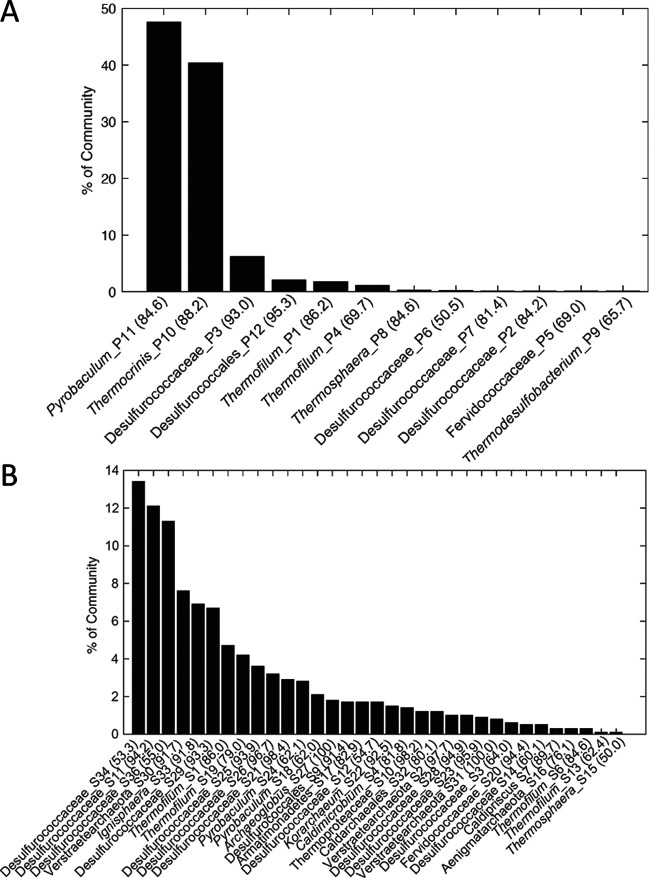
Rank-abundance plot of Gibbon Geyser Spring 063 (GGS063) metagenome-assembled genomes (MAGs) from planktonic (A) and sediment (B) communities. The relative abundance of MAGs with an estimated completeness of >50% is presented (MAG completeness indicated in parentheses). MAG taxonomy was assessed using GTDB-Tk classification and reported with the average nucleotide identity (ANI) to the highest taxonomic rank available, as reported in Table S2.

A total of 12 and 33 MAGs were recovered from the planktonic and sediment communities in GGS063, respectively, indicating that the latter is taxonomically richer than the former. These observations were also highly consistent with 16S rRNA gene diversity observed within the entire metagenomes (16 versus 28 operational taxonomic units, respectively). Consistent with the planktonic community being less taxonomically rich, it was dominated by two MAGs, one of which is closely related (95.2% average nucleotide identity [ANI]) to the archaeon Pyrobaculum yellowstonensis WP30 (bin P11; 47.6% of community) and the other that is closely related (93.4% ANI) to the bacterium Thermocrinis ruber OC 1/4 (P10; 40.4%) (Fig. 2A; Table S2). The type strains of T. ruber OC 1/4 and P. yellowstonensis WP30 were both isolated from circumneutral to alkaline hot springs in YNP (12, 18), and close relatives are commonly detected by molecular analyses of springs sourced by the parent hydrothermal aquifer in YNP (13, 18, 39, 40). In contrast to the planktonic community that consists of both bacteria (40.5% of community) and archaea (59.5%), MAGs recovered from the sediment community were primarily affiliated with archaea (96.6%), and their abundance was more evenly distributed (Fig. 2). Moreover, the combined abundances of the top 17 most abundant MAGs in the sediment (88.3% of community) were nearly equivalent to the combined abundance (88.0% of the community) of the two most abundant MAGs in the plankton community (Fig. 2). The most abundant MAG in the sediment community (bin S34; 13.3% of community) was distantly related to the archaeon Desulfurococcus amylolyticus (76.3% ANI) (Fig. 2B). The distinctly different taxonomic structures of the GGS063 planktonic and sediment communities suggest different factors are influencing the assembly of these two communities. The dominance of the planktonic community by two taxa, *Pyrobaculum* (bin P11) and *Thermocrinis* (P10), suggests that these taxa are efficient at acquiring nutrients from the water column to fuel their metabolism and growth. Dominance of the planktonic community suggests that the spectrum of nutrients available in the water column is narrow, allowing for minimal partitioning of niche space to support additional abundant taxa (41). In contrast, the more even distribution in the abundances of MAGs in the sediment community suggests that the greater diversity of taxa may be supported by a wider spectrum of available electrons and acceptors that may include minerals (e.g., elemental sulfur), as has been suggested previously for hot spring ecosystems supported by chemical energy (13). It is equally plausible that the apparent prevalence of anaerobes in the sediment community can be attributed to those organisms being excluded from the water column community due to their sensitivity to O_2_.

A common characteristic of MAGs comprising both the planktonic and sediment communities is that they are distantly related to cultivars. For the planktonic community, only 2 of 8 MAGs (ANIs could not be calculated for 4 MAGs due to incompleteness) exhibited more than 90% ANI to a cultured species, with the remainder of MAGs exhibiting ANIs to characterized species that ranged from 75 to 90% (Table S2). This trend is even more pronounced in the sediment community, with only 7 of the 25 MAGs (ANIs could not be calculated for 9 MAGs due to incompleteness) exhibiting more than 90% ANI to a characterized species. These observations could be explained by the fact that most studies of circumneutral to alkaline hot springs in YNP have been focused on biofilm (i.e., “streamers”) (4, 39, 42), and fewer have been conducted on communities inhabiting the water columns and sediments of these springs. These observations call for additional cultivation-dependent and cultivation-independent characterizations of populations inhabiting circumneutral to alkaline springs.

### Phylogenetic similarity analysis.

**FIG 3:**
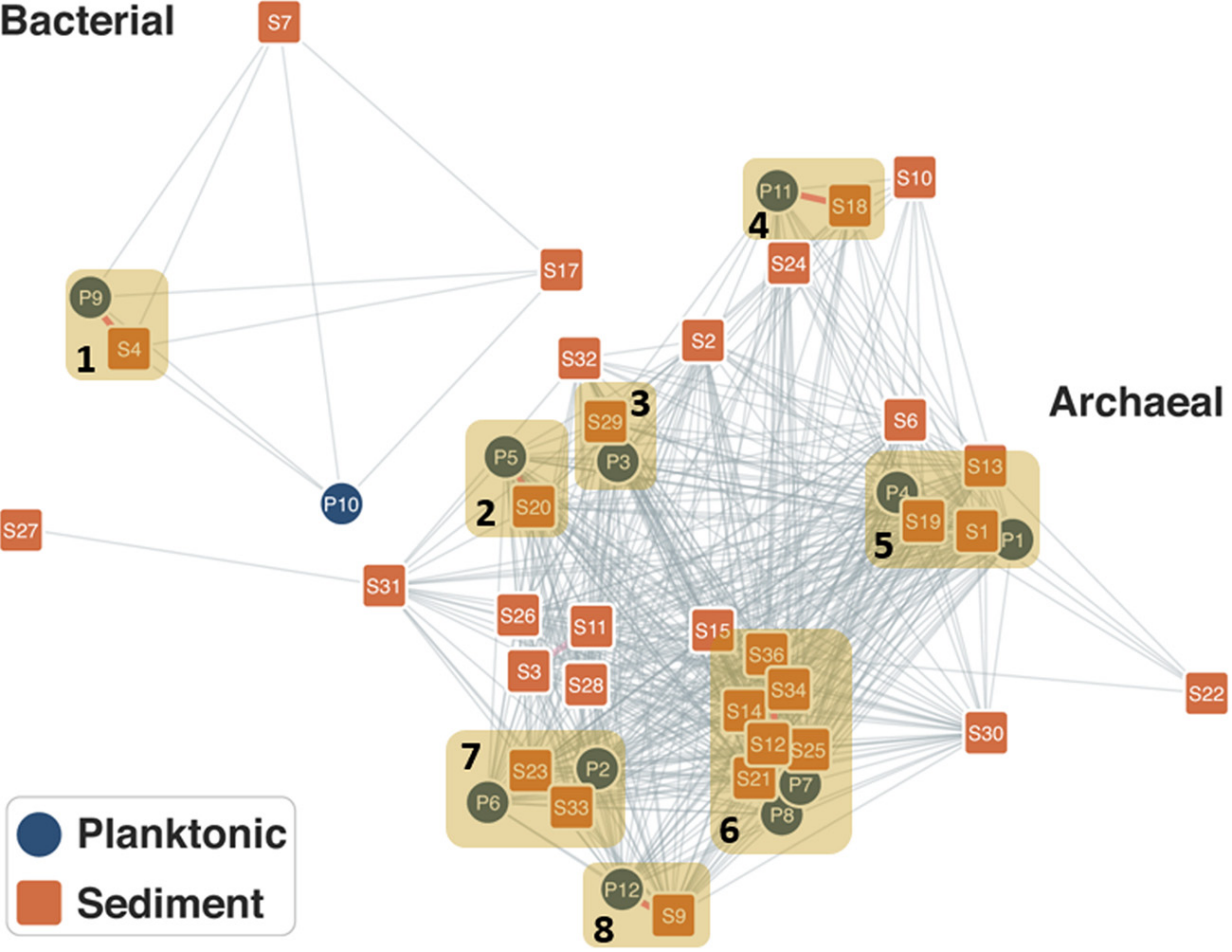
Correlation network of 45 archaeal and bacterial MAGs (nodes) from Gibbon Geyser Spring 063 (GGS063). The network depicts the average amino acid identity (AAI) of MAGs, with longer edges indicating lower AAI values and shorter edges indicating higher AAI values. Edges are only shown between pairs of MAGs when AAI is >40%. Edge colors in pink denote AAI of >90%. The node designations correspond to the bins in Fig. 2 and as defined in Table S2. Node colors in orange denote MAGs recovered from GGS063 sediment (sediment community), and nodes in blue denote MAGs recovered from the GGS063 water column (planktonic community). Clusters of nodes are numbered 1 to 8 and correspond to those discussed in the text.

A network was built from an amino acid identity (AAI) matrix depicting the similarities among the 45 sediment and planktonic MAGs from GGS063 (Fig. 3). The sediment MAGs (nodes), which are predominantly archaeal, define the topology of the network, a finding that is consistent with the observation that nearly all planktonic MAGs are connected by short branches to a closely related sediment MAG. The only exception is the *Thermocrinis* MAG (bin P10) that does not have a corresponding high-quality MAG in the sediment community. Eight predominant MAG clusters were identified in the network, including MAGs affiliated with the *Thermodesulfobacteriaceae* (cluster 1; bins P9 and S4; pairwise AAI, 98.1%), *Fervidococcaceae* (cluster 2; P5 and S20; pairwise AAI, 98.2%), *Desulfurococcaceae* (cluster 3; P3 and S29; pairwise AAI, 99.4%), *Pyrobaculum* (cluster 4; P11, S18, and S24; mean pairwise AAI, 89.1%), *Thermofilaceae* (cluster 5; P1, P4, S1, S6, S13, and S19; mean pairwise AAI, 64.0%), *Desulfurococcaceae* (cluster 6; P7, P8, S12, S14, S15, S21, S25, S34, and S36; mean pairwise AA, 67.6%), *Ignisphaera* (cluster 7; P2, P6, S23, and S33; mean pairwise AAI, 72.8%), and *Desulfurococcales* (cluster 8; 12P and 9S, mean pairwise AAI, 98.9%). These clusters comprise closely related MAGs that were identified in both the sediment and planktonic communities. Within a defined cluster, the sediment-plankton MAG pairs tend to have a higher pairwise AAI than MAGs from within the same community; planktonic MAGs do not form a cluster of their own. Collectively, this observation supports the results from the rank abundance analysis, indicating that the planktonic community is taxonomically dominated by a few phylotypes, whereas the sediment community is taxonomically more even and is comprised of a more diverse array of closely related phylotypes.

The observation that the sediment community in GGS063 is more evenly structured and more biodiverse than the planktonic community is consistent with a previous study of sediment and planktonic communities in 15 high-temperature hot springs in YNP (13). In that study, it was hypothesized that the higher diversity and evenness in sediment communities, compared to planktonic communities, can be attributed to those communities being differentiated by characteristics of their local habitat (13). Specifically, based on 16S rRNA gene inference to cultivars, it was suggested that planktonic communities are supported to a greater extent by aerobic metabolisms, whereas sediment communities are largely supported by mineral-based anaerobic metabolisms, in particular, those involving elemental sulfur or ferric iron reduction. This was interpreted to reflect differential availability of O_2_, the preferred electron acceptor for the metabolisms of thermophiles (3, 8), in the hot spring water column versus the sediments. This motivated the hypothesis that planktonic and sediment populations in GGS063 are differentiated by oxidant availability, in particular, dissolved O_2_, and the metagenomics-enabled functional analysis of the encoded potential of planktonic- and sediment-associated organisms to respire O_2_, which was tested herein.

### Metabolic differentiation of planktonic and sediment MAGs.

**FIG 4:**
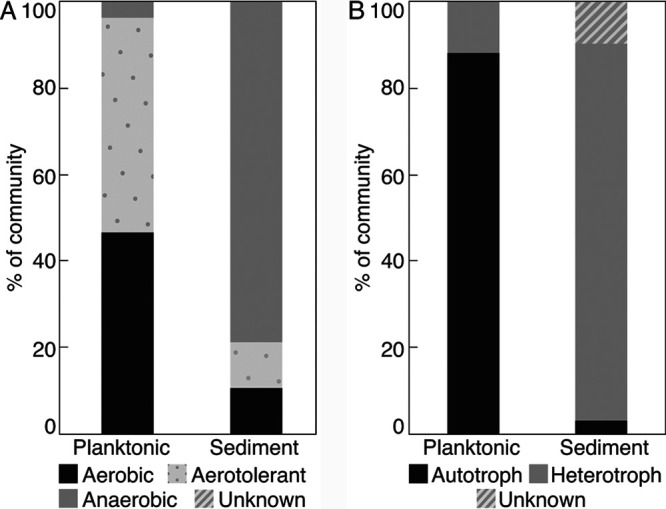
Inferred metabolic potential of planktonic and sediment communities from Gibbon Geyser Spring 063 (GGS063). (A) The predicted ability for a population to integrate oxygen into their energy metabolism, as assessed by proteins encoded in MAGs comprising those communities, is depicted. MAGs were classified as aerobic, anaerobic, or aerotolerant using criteria outlined in the text. Oxygen preference for *Thermofilum* (bin P4) and *Aenigmatarchaeota* (S16) could not be determined and are labeled as unknown. (B) Predicted ability for a population to perform autotrophy in the planktonic and sediment communities from GGS063. Populations were designated autotrophs or heterotrophs based on proteins encoded in MAGs. The mode of carbon metabolism of *Verstraetearchaeota* (bins S2, S30, and S31) and *Aenigmarchaeota* (S16) could not be determined and are labeled as unknown.

Roughly 47% and 50% of the planktonic community members were predicted to be capable of aerobic metabolism or to be aerotolerant, respectively, totaling roughly 97% of the community (Fig. 4A). This includes two abundant MAGs most closely related to *Thermocrinis* (bin P10; 40.4% of the community) and *Desulfurococcaceae* (P3; 6.2%) that both encode homologs of cytochrome *c* oxidase (Cox). The most abundant MAG in the planktonic community that is related to *Pyrobaculum* (P11; 47.6%) encodes a homolog of cytochrome *bd* oxidase (CydA), which is common in P. yellowstonensis WP30 and other *Crenarchaeota*. No members of *Pyrobaculum* are known to respire O_2_ using CydA, and this MAG is thus predicted to correspond to an aerotolerant anaerobe. Like P. yellowstonensis WP30 (18), the GGS063 *Pyrobaculum* P11 MAG encodes several homologs of dimethyl sulfoxide (DMSO) reductase that are inferred to function in elemental sulfur/polysulfide reduction and a homolog of arsenate reductase (ArrA) that is inferred to function in arsenate reduction, suggesting that elemental sulfur or As(V) may be respired by this organism. Interestingly, this MAG also encodes a homolog of [NiFe] hydrogenase and 2 homologs of arsenite oxidase (AioA), suggesting that it can use H_2_ or As(III) as electron donors. This would potentially help to explain how this strain could grow autotrophically via the dicarboxylate/4-hydroxybutyrate (DC/4HB) pathway (discussed below). Similar to *Pyrobaculum* bin P11, a MAG most closely related to *Thermofilum* (P1; 1.8%) is predicted to be aerotolerant based on it encoding CydABX. The detection of abundant *Pyrobaculum-* and *Thermofilum*-affiliated MAGs that are predicted to be aerotolerant anaerobes in GGS063 is consistent with the limited solubility of O_2_ in nearly boiling waters (boiling point is 93°C in YNP) of GGS063 (85.9°C, when sampled) (31) (Fig. 1C). Based on the detection of homologs of AioA, sulfide-quinone oxidoreductase (Sqo), and thiosulfate/sulfur oxidase (Sox), T. ruber is inferred to be capable of utilizing As(III), sulfide, or the sulfide oxidation product thiosulfate as an electron donor during O_2_ respiration (discussed below). It is thus possible that aerobic respiration by T. ruber in the water column of GGS063 facilitates the presence of *Pyrobaculum* and *Thermofilum* by consuming O_2_. In the case of *Pyrobaculum*, T. ruber may also be a source of elemental sulfur/polysulfide through incomplete oxidation of sulfide or As(V) via oxidation of As(III). As such, only 3.9% of the planktonic community was determined to be comprised of strict anaerobes.

In contrast to the planktonic community that is primarily comprised of putative aerobes or aerotolerant anaerobes, 23 out of the 33 sediment MAGs, which represent ∼79% of the community, are predicted to be strict anaerobes, including the 5 most abundant MAGs, *Desulfurococacceae* (bin S34; 13.4% of the community), *Desulfurococacceae* (S11; 12.1%), *Desulfurococacceae* (S36; 11.3%), *Verstraetearchaeota* (S30; 7.6%), and *Ignisphaera* (S33; 6.9%). The detection of divergent homologs of sulfite/tetrathionite reductase (AsrAB) in *Desulfurococacceae* bins S34 and S36 and *Ignisphaera* S33 suggests the possibility that these populations reduce an oxyanion of sulfur (e.g., sulfite, tetrathionate) that might be produced in the water column through incomplete oxidation of sulfide (e.g., *Thermocrinis*). *Desulfurococacceae* bin S11 likely corresponds to an anaerobic heterotroph or fermenter, based on the lack of homologs allowing for other anaerobic respiratory capabilities and the lack of homologs of autotrophic pathways (described below). It is also possible that such proteins are encoded in the genome of S11 but either were not sequenced or did not assemble well. Like many environmental *Verstraetearchaeota* MAGs recovered from hot springs and other environments (43), all three bins (S2, S30, and S31) from GGS063 encode enzyme homologs involved in anaerobic methane or alkane metabolism (methanogenesis, methanotrophy, alkanotrophy), but only one bin (S31) encodes methyl CoM reductase subunit A (McrA), a genetic marker for these processes. Two of the three bins (S2, S30) encoded homologs of [NiFe] hydrogenase. Thus, the metabolism of these organisms is not clear, but they may be supported by oxidation of H_2_ or oxidation of methane or other alkanes by yet-to-be-defined pathways. Another six sediment MAGs (11.7% of the community) are predicted to be aerotolerant based on detection of CydABX, including *Thermofilum* (bin S1; 4.7% of community), *Pyrobaculum* (S18; 2.1%), *Archaeoglobus* (S27; 1.8%), *Korarchaeum* (S22; 1.5%), and *Caldimicrobium* (S4; 1.4%). Only three MAGs are predicted to be aerobic based on detection of Cox, and a single MAG could not be confidently assigned as aerobic, aerotolerant, or anaerobic (S16; *Aenigmatarchaeota*). The two aerobic MAGs with greater than 1% abundance belong to uncharacterized *Desulfurococacceae* (bin S29; 6.7% of community) and *Armatimonadetes* (S17; 1.7%).

The dichotomy in the predicted metabolism of O_2_ or its detoxification in the planktonic community (∼97% of the community correspond to organisms capable of respiring or detoxifying O_2_) versus the sediment community (∼79% of the community correspond to strict anaerobes) suggests that the availability of O_2_ likely varies markedly between these two environment types within the hot spring. The waters sourcing GGS063 are deficient in O_2_ due to its consumption via water-rock interactions during the lengthy inferred residence time (>200 years) (26–28) that these waters have been out of contact with the atmosphere. Further, the geochemical composition of GGS063 waters reveals little evidence for mixing with more oxidized near-surface water (e.g., nitrate below detection, no evidence for dilution of sulfate/chloride). This is possibly due to silica armoring the conduit(s) feeding this spring (23, 24) and thus restricting near-surface inputs of groundwater. To the extent this is true and since the temperature of GGS063 is too high for oxygenic photosynthesis (6, 7), the primary source of oxygen in GGS063 waters is likely influx from the atmosphere, which provides limited O_2_ in waters with high temperature (85.9°C) (31), such as those in GGS063. It is suggested that the high proportion of anaerobes in the sediment community is thus due to limited availability of O_2_ in GGS063 (0.021 mg liter^−1^) due to limited influx of atmospheric gases combined with consumption by aerobes (e.g., T. ruber) comprising the planktonic community that render the sediment environment of the spring suboxic or anoxic, allowing for or facilitating habitation by anaerobes. The low concentration of dissolved O_2_ in waters of GGS063 (0.021 mg liter^−1^) is similar to other springs in YNP sourced by the parent hydrothermal aquifer (<0.042 mg liter^−1^) (4), suggesting that the dichotomy between planktonic and sediment communities as it relates to O_2_ usage is unlikely to be limited to GGS063.

A previous study of 181 hot springs in YNP showed that circumneutral to alkaline springs sourced with parent hydrothermal water also tend to be limited in DOC compared to those that are sourced by mixed meteoric and hydrothermal fluids (25). The low concentration of DOC (0.4 mg liter^−1^) in water from GGS063 (Fig. 1) is consistent with this previously observed pattern. Like O_2_, the limited availability of DOC in circumneutral to alkaline springs may be attributable to silica armoring of the conduit(s) feeding this spring type that may prevent substantial input of near-surface DOC-enriched ground- or surface waters (44). Further support that circumneutral to alkaline springs tend to be limited in autochthonous DOC comes from isotopic analyses of streamer biofilms from several other alkaline springs across YNP (42, 45). These studies suggest the streamer biofilm communities primarily comprise autotrophs, but these populations are facultative and rapidly (albeit temporally) switch to heterotrophic or mixotrophic metabolisms when allochthonous organic carbon is input into springs from runoff due to summer rainstorms. Since GGS063 is sourced by deep hydrothermal parent fluids and has low concentrations of DOC, it was thus hypothesized that microbial populations at the time of sampling (July 2017) would be capable of autotrophic metabolism. Like the streamer biofilm communities, it is possible that these autotrophs are facultative and would switch to heterotrophic metabolism should allochthonous organic carbon become temporally available.

To examine this hypothesis, the inferred proteomes of GGS063 planktonic and sediment MAGs were examined for the key proteins associated with the six known autotrophic pathways (46, 47). These included (i) the Calvin-Benson-Bassham (CBB) cycle, (ii) the reductive tricarboxylic acid (rTCA) cycle, (iii) the Wood-Ljungdahl (WL) pathway, (iv) the 3-hydroxypropionate (3HP) bicycle, (v) the 3HP/4HB cycle, and (vi) the DC/4HB cycle. Similarly, the proteomes of MAGs were examined for proteins that would allow for the use of other inorganic electron donors and acceptors that could potentially inform on their ecology in GGS063.

Roughly 88.1% of the planktonic community is predicted to be capable of autotrophic metabolism (Fig. 4; Table S7). This includes MAGs closely related to *Pyrobaculum* (bin P11; 47.6% of the community), *Thermocrinis* (P10; 40.4%), and *Thermodesulfobacterium* (P9; 0.1%) that encoded the genetic potential to be autotrophic. Specifically, *Pyrobaculum* encoded the DC/4HB pathway, *Thermocrinis* encoded the rTCA cycle, and *Thermodesulfobacterium* encoded the WL pathway. *Pyrobaculum* (P11) is inferred to respire elemental sulfur/polysulfide or As(V) based on the MAG encoding a variety of DMSO reductase homologs and a homolog of arsenate reductase, ArrA. Electron donors may include H_2_ or As(III) based on the MAG encoding homologs of [NiFe] hydrogenase and arsenite oxidase, AioA. *Thermocrinis* (P10) is predicted to support autotrophic growth by coupling the oxidation of sulfide (homologs of Sqo), thiosulfate (Sox), or As(III) (AioA) to the reduction of O_2_ (Cox). Finally, *Thermodesulfobacterium* (P9) is predicted to support autotrophic growth by coupling H_2_ oxidation (homologs of [NiFe] hydrogenase) to SO_4_^2−^ reduction (homologs of Sat, AprAB, DsrAB). *Thermodesulfobacterium* may supplement autotrophic metabolism with organic carbon in a mixotrophic mode of metabolism or may be facultatively autotrophic, as evidenced by the MAG encoding a full glycolysis pathway. It is important to note that P. yellowstonensis WP30, which also encodes the DC/4HB pathway, could not be cultivated autotrophically when grown anaerobically on elemental sulfur or As(V) (18). However, many archaea, including sulfur- and iron-reducing archaea (48, 49), are known to harbor mixotrophic carbon and energy metabolisms, and mixotrophic metabolism was not specifically examined in the study of P. yellowstonensis WP30.

Surprisingly, only two sediment MAGs comprising just 3.2% of the community encoded autotrophic pathways (Fig. 4; Table S7) This included MAGs most closely related to the bacterium *Caldimicrobium* (bin S4; 1.4% of the community) and the archaeon *Archaeoglobus* (S27; 1.8%), both of which encoded the WL pathway. *Caldimicrobium* and *Archaeoglobus* are predicted to couple H_2_ oxidation (homologs of [NiFe] hydrogenases) to SO_4_^2−^ reduction (homologs of DsrAB, Sat, and AprAB) to fuel autotrophic growth. Both the *Caldimicrobium* and *Archaeoglobus* MAGs also encode a complete glycolysis pathway, indicating the organisms are facultative autotrophs, and the *Archaeoglobus* MAG encodes a complete TCA cycle.

Only 11.9% of the planktonic community is inferred to be obligately heterotrophic (Fig. 4; Table S7), based on the absence of genes in MAGs that encode autotrophic pathways. Of these putative heterotrophic MAGs, *Desulfurococcaceae* (bin P3; 6.2% of the community) is inferred to be an aerobic heterotroph based on the presence of homologs of Cox. Aerobic members of the *Desulfurococcaceae* are rare (50), however, this MAG shares 79.3% DNA-directed RNA polymerase subunit B (RpoB) identity with that of Aeropyrum camini (*Desulfurococcaceae*), which is capable of aerobic respiration (51). All other planktonic heterotrophic MAGs (*Desulfurococcales*, bin P12; *Thermofilum*, P1; *Thermofilum*, P4; *Desulfurococcaceae*, P6, P2, and P7; *Fervidococcaceae*, P5; and *Thermosphaera* in P8) are inferred to be obligately fermentative due to the apparent absence of terminal reductases and the presence of homologs of enzymes that typify fermentative organisms, such as alcohol dehydrogenases.

In contrast to the planktonic community, inferred heterotrophs constituted 87.2% of the sediment community, suggesting organic carbon serves as both carbon source and electron donor for these populations (Fig. 4; Table S7). Based on the inferred proteomes of MAGs, four strategies of heterotrophic metabolism were observed: fermentation or heterotrophic respiration of O_2_, As(V), or a variety of sulfur compounds. Several heterotrophic MAGs in the sediment, including *Desulfurococcaceae* (bins S11, S14, S21, S26, S28, S34, and S36), *Pyrobaculum* (S24), and Thermoproteaceae (S10), encoded the potential for dissimilatory SO_4_^2−^ (homologs of DsrAB, AprA, Sat) and/or sulfite/tetrathionate (Asr) reduction. Heterotrophic MAGs predicted to be aerobes (homologs of Cox) included *Desulfurococcaeae* (bin S29), *Armatimonadetes* (S17), and *Candidatus* Caldipriscus (S7). All other sediment heterotrophic MAGs, including *Ignisphaera* (bin S33), *Thermofilum* (S1, S6, S13, and S19), *Desulfurococcaeae* (S3, S12, and S25), *Desulfurococcales* (S9), *Caldarchaeales* (S32), *Ignisphaera* (S23), *Fervidococcaceae* (S20), and *Thermosphaera* (S15) are inferred to be fermentative due to the apparent absence of terminal reductases and the presence of homologs of fermentative enzymes such as alcohol dehydrogenases. It is important to note that *Pyrobaculum* (bin S24) encoded many of the enzymes required for the DC/4HB pathway but did not encode a key enzyme demarcating this pathway, 4-hydroxybutyrate dehydrogenase. It is possible that this is due to the relatively low completeness (62.1%) of the genome. Nonetheless, given that it represents only 2.8% of the community, referring to this genome as autotrophic or heterotrophic would not markedly change the observation that the majority of the sediment community is comprised of putative obligate heterotrophs.

### Genome replication rate estimations.

Estimates of genome replication rates (i.e., GRiD scores) were calculated for MAGs with >50% completeness and <5% contamination (Table S2). However, since phylogenetically distinct organisms may replicate their genomes at different rates independent of environmental characteristics or growth status (52), only GRiD scores calculated for pairs of phylogenetically similar MAGs in planktonic and sediment communities were compared. Planktonic and sediment MAGs that shared >95% ANI were identified (eight MAG pairs in total) (Table S4) and their GRiD scores at the time of sampling were estimated and compared (Table S7). These eight MAG pairs include those related to *Thermofilum* (bins P1 and S1), *Pyrobaculum* (P11 and S18), *Desulfurococcales* (P12 and S9), *Fervidococcaceae* (P5 and S20), *Desulfuroccocaceae* (P6 and S23), *Desulfuroccocaceae* (P7 and S25), *Thermosphaera* (P8 and S36), and *Thermodesulfobacteraceae* (P9 and S4). All of these pairwise comparisons involve MAGs from putative aerotolerant anaerobes or strict anaerobes; the MAGs from putative aerobes either did not have a closely related MAG of high quality in the sediment community (i.e., *Thermocrinis* bin P10), or one of the pairwise MAGs did not meet quality standards for completeness or for *dif*/*ter* ratios (i.e., *Aeropyrum* P3 and S29).

**FIG 5:**
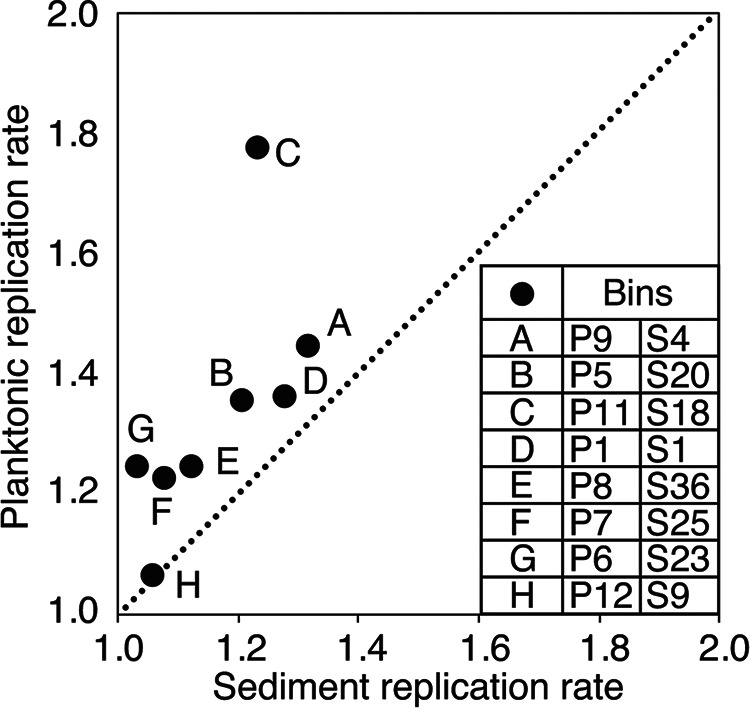
Estimated replication rates (GRiD scores) for pairs of metagenome assembled genomes (MAGs) from the planktonic and sediment communities that exhibit average nucleotide identity (ANI) and amino acid identity (AAI) exceeding 95%. Each point represents a comparison of a MAG pair from planktonic and sediment communities, as defined in the legend. A 1:1 line representing equivalent GRiD scores for each pairwise comparison is shown. Bin designations are as presented in Fig. 2 and Table S2. GRiD scores for planktonic and sediment MAGs are presented in Table S7.

In seven of eight pairwise comparisons, the GRiD scores for planktonic MAGs exceeded those of the sediment MAGs (Fig. 5A). In particular, the *Pyrobaculum* population in the planktonic community (bin P11) had a substantially higher GRiD score than the corresponding MAG in the sediment (S18). One MAG pair, *Desulfurococcales* (P12 and S9), had a higher, albeit not statistically significant, GRiD score in the sediment community than the planktonic community. This suggests that GRiD scores in phylogenetically closely related MAGs were higher in the plankton community than in the sediment community. To the extent that the pattern of higher genome replication rates for phylogenetically closely related MAGs of aerotolerant anaerobes and strict anaerobes in the planktonic versus the sediment community extends to MAGs of aerobes, these results indicate that populations comprising the planktonic community are likely replicating their genomes at a higher rate than are those of the sediment community. This is true even for populations (i.e., *Pyrobaculum*) with autotrophic capabilities that would add to the metabolic burden of the cell. Estimates of GRiD calculations for all MAGs are reported in Table S7.

Higher inferred genome replication rates among planktonic versus sediment populations may reflect increased availability of high potential oxidants to support their energy metabolism and biomass generation. Based on metabolic inferences and geochemical data, one may predict that this is due to the planktonic populations having better access to O_2_ (e.g., *Thermocrinis*) and As(V) (e.g., *Pyrobaculum*). Access to higher concentrations of such substrates, when combined with available electron donors such as sulfide/thiosulfate (e.g., *Thermocrinis*), As(III) (e.g., *Thermocrinis*), and H_2_ (e.g., *Pyrobaculum*), could provide the energy necessary to support autotrophic growth that then could support secondary production from other heterotrophic members of the community, including those that reside in the water column or in the sediment of GGS063. The higher inferred rates of genome replication for planktonic populations compared to sediment populations, when combined with the inferred autotrophic capabilities of the planktonic populations and heterotrophic capabilities of the sediment populations, is potentially consistent with a trophic level pyramid, if the autotrophic populations are cross-feeding heterotrophic populations.

**Conclusions.** Data presented herein from GGS063, a hot spring with a chemical composition consistent with it being sourced from the parent hydrothermal aquifer, reveal extreme dichotomy in the taxonomic composition and inferred metabolic potential of planktonic and sediment communities. Both planktonic and sediment communities are taxonomically representative of those identified in other circumneutral to alkaline hot springs, suggesting the observations made here could extend beyond GGS063. The planktonic and sediment communities in GGS063 were dominated by archaea, a finding that is unique among circumneutral to alkaline communities studied to date, likely due to the focus of previous studies on streamer biofilms that are dominated by the bacterium *Thermocrinis* (4, 39, 42). Further, the planktonic community is predominantly comprised of organisms capable of respiring or detoxifying O_2_ that are also capable of autotrophic growth, while the sediment community consists predominantly of anaerobes capable of heterotrophic growth. The prevalence of organisms capable of autotrophy is consistent with the limited amounts of DOC in hot springs sourced by the parent hydrothermal aquifer (25), including GGS063. The prevalence of putative aerobes or aerotolerant anaerobes in the planktonic community is interpreted to reflect influx of atmospheric O_2_ to otherwise anoxic hydrothermal waters sourcing GGS063. The higher estimated genome replication rates in the planktonic community are attributed to greater access to nutrients due to turbulence in the water column as well as a greater availability of high-potential electron acceptors, such as O_2_ from ingassing.

The apparent role of ingassing of O_2_-rich atmospheric gas in driving the dichotomy in the taxonomic composition and inferred metabolic potentials of planktonic and sediment communities in this and other circumneutral to alkaline springs across YNP (13) indicates that the ecology of this spring type may have changed markedly over Earth’s history. While this type of hot spring is often considered an analog for early Earth environments (53, 54), the apparent effects of O_2_ on the ecology of planktonic populations in GGS063, and potentially other circumneutral to alkaline springs in YNP and elsewhere, raise the question of how well these contemporary communities and their functionalities reflect those that inhabited such springs prior to the rise of O_2_ in the atmosphere ∼2.4 Gya (55). Further research is thus warranted to determine if other springs sourced by anoxic waters from the parent hydrothermal aquifer host dichotomous microbial communities and to establish the extent that such springs can serve as early Earth analogs.

## MATERIALS AND METHODS

### Sample collection and geochemical analyses.

An unnamed hot spring (YNP thermal inventory identifier GGS063; lat 44.69047; long −110.72917; elevation, 2,291 m) in the northern Geyser Creek Group, Gibbon Geyser Basin, YNP, was selected for study and sampled on 21 June 2017. The temperature, conductivity, and pH of GGS063 were measured on-site with temperature-compensated probes (model YSI EC300; YSI Inc., Yellow Springs, OH, USA or model WTW 3100, WTW Weilheim, Germany). Dissolved Fe(II) and total sulfide (S^2−^) concentrations were measured in the field using Hach reagents (ferrozine pillows and sulfide reagents 1 and 2) and a Hach DR/890 spectrophotometer (Hach, Loveland, CO). Dissolved oxygen was measured *in situ* using a fluorescence method with a detection limit of 15 ppb and a resolution of ±4 ppb (Fibox 4 m and optical probe; PreSens Inc., Germany) following methods of St. Clair et al. (56). Dissolved oxygen measurements were made at ∼20 cm below the air-water interface. Spring waters were filtered (0.22 μm) and stored at 4°C for determination of anions via ion chromatography (Metrohm Compact Plus; Herisau, Switzerland), cations via inductively coupled plasma optical emission spectrometry (Thermo Scientific iCAP 6000 series; Waltham, MA), trace metals via inductively coupled plasma mass spectrometry (Thermo Scientific iCAP Q), and dissolved organic carbon via an OI Analytical wet oxidation total organic carbon analyzer coupled to a Thermo Delta Plus Advantage mass spectrometer as previously described (57) based on the methods of St-Jean (58). Anions, cation, and trace metal determinations were made at the Analytical Lab at the Montana Bureau of Mines and Geology.

Triplicate subsamples of sediment (0.5 g each) were collected from GGS063 using a flame-sterilized spatula and were transferred to sterile 1.5-ml microcentrifuge tubes. Planktonic biomass was collected from a depth of ∼20 cm beneath the atmosphere-water interface in the hot spring with a peristaltic pump (Geotech Environmental Equipment, Denver, CO) and sterilized tubing prerinsed with ∼10 liters of hot spring water. Fluids were pumped through sterile 0.2-μm 47-mm Supor polyethersulfone (PES) membrane disc filters (Pall Corporation, Port Washington, NY) held in autoclaved in-line steel filter housings (Pall Corp.) in triplicates of 8 liters each. After filtering, filters were removed from the housings using flame-sterilized forceps and transferred aseptically into sterile 50-ml Falcon tubes. The 1.5-ml microcentrifuge tubes containing sediments and the 50-ml tubes containing filters were then placed on dry ice for transport to the lab, where they were kept at −80°C.

DNA was extracted from filtered biomass and sediment-associated biomass with the FastDNA Spin Kit for Soil (MP Biomedicals, Irvine, CA) following the manufacturer’s instructions. Genomic DNA was quantified fluorometrically via the high-sensitivity Qubit assay (Thermo Fisher Scientific, Waltham, MA) and was subjected to paired-end shotgun metagenomic sequencing (Illumina NovaSeq; 2 × 151 bp) at the Department of Energy Joint Genome Institute (JGI; Walnut Creek, CA) following the Illumina regular fragment (300 bp) method of library preparation (59). Reads were quality filtered, trimmed, and cleaved of Illumina-specific sequencing adapters using BBTools and then read corrected using bbcms (within BBTools) via the standard JGI metagenome processing pipeline. Reads were then assembled into contigs using metaSPAdes v.3.13.0. Metagenome assembly statistics are reported in Table S1 in the supplemental material. The assembled contigs were binned into metagenome-assembled genomes (MAGs) using the MetaWRAP platform (60) to map reads to contigs using BWA aligner v.0.7.17 (61). MetaWRAP was also used to generate MAGs with the MetaBAT v.2.12.1 (62), MaxBin v.2.0 (63), and CONCOCT v.1.0.0 (64) binning programs and contigs >2,500 bp. Finally, MetaWRAP was used to identify the best-quality MAGs from the three aforementioned binning strategies via “contamination” and “completion” estimates from CheckM v.1.1.3 (65) and the bin_refinement module. Only MAGs that exhibited >50% estimated completeness and <10% contamination (consistent with moderate- to high-quality genomes [66]) were retained for further analyses.

Binned contigs were mapped back to the JGI scaffolds. To evaluate potential abundances of populations within communities, the BWA-mapped reads to contigs data set was summed across binned MAGs using the “profile” module within CheckM. The reported relative abundances are based on overall number of reads mapped to individual MAGs, then corrected for estimated MAG sizes, and ultimately reported as a proportion of all binned MAGs for each metagenome. The MAGs were taxonomically classified using the GTDB-Tk v.1.3.0 (67) classifier and the bac120 and ar122 data sets for bacterial and archaeal classification, respectively. Taxonomic designations are given to the lowest taxonomic rank that is formally characterized. The planktonic (JGI Integrated Microbial Genomes [JGI] name, GCR.EP_P) and sediment (IMG name, GCR.EP_S) metagenomes from GGS063 are available on JGI-IMG under genome identifiers 3300033431 and 3300033490, respectively.

### Comparison of GGS063 16S rRNA gene compositions to those of other circumneutral hot springs in YNP.

To evaluate the similarity of GGS063 communities to those of other circumneutral hot springs in YNP, 16S rRNA genes were retrieved from the IMG database for both the sediment and planktonic communities from GGS063 and compared against sediment and planktonic community compositions of 15 other hot springs previously reported in Colman et al. (13). Briefly, the metagenome-derived 16S rRNA genes were subject to alignment to the Silva reference database with operational taxonomic units (OTU) representatives from the other 30 communities, trimmed to the length of the V4-V5 hypervariable region used in the previous study, clustered into OTUs at the 97% nucleotide sequence identity level, and classified against the Silva database, all as described previously (38). The GGS063 OTUs were added to those from the previous data set based on clustering with the previously identified representative 16S rRNA gene sequences. Finally, the GGS063 OTUs were ascribed relative abundances based on the relative sequencing depth coverage values of the metagenomic contigs from which they derive. These data were then used to construct nonmetric multidimensional scaling (NMDS) ordinations inclusive of the GGS063 plankton and sediment 16S rRNA communities, as well as those from Colman et al. (13), as described in the latter study.

### Phylogenetic similarity analysis.

To visualize the phylogenetic relationships among MAGs recovered from the sediments and plankton communities, whole-genome pairwise amino acid identity (AAI) values were calculated with the AAI comparison workflow (aai_wf module) within the CompareM v.0.0.23 software package (http://github.com/dparks1134/CompareM) with default parameters for protein comparisons (minimum identity of 30%, minimum alignment length of comparison query of 70%). The pairwise AAI values were then used as edges within the network visualization tool, Cytoscape v.3.8.2 (68). The force-directed edge-weighted layout was used to structure MAG positions based on their among-MAG AAI values while only considering pairwise values >40%.

### Metabolic potential of MAGs.

The amino acid sequences of MAGS were uploaded to the Kyoto Encyclopedia of Genes and Genomes (KEGG) function database (69) with the KEGG Automatic Annotation Server (KAAS) (70). Annotations were mapped through bidirectional best hit (BBH) searches against KAAS system metabolic pathways. Initial inferences of metabolic capability were made based on KEGG Orthology (KO) mapping to the “oxidative phosphorylation” and “carbon fixation of prokaryotes” pathway groups. To explore additional functional potential, a type strain or closely related genome was identified for each MAG based on the relatedness of the housekeeping proteins DNA-directed RNA polymerase subunit b (RpoB) or the 50S ribosomal protein L2 (RPL2). Demonstrated metabolic capabilities from the related cultivars with available genomes guided further metabolic reconstruction analyses. Sequences of interest from these genomes were then used as queries for Basic Local Alignment Search Tool (BLASTp) (71) searches against MAGs using the National Center of Biotechnology Information (NCBI) tool. An E value cutoff of 1.0e^−20^ was used to screen homologs along with >30% amino acid identity and >60% coverage of the query sequence. Annotations of consequential sequences from both KO mapping and BLASTp analyses were verified by structural homology using SWISS-MODEL (72) and/or by alignment in Clustal Omega (v.1.2.4) (73) to identify motifs or residues that are required for functionality.

The potential for planktonic and sediment-associated populations in GGS063 to integrate O_2_ into their energy metabolism was assessed based on MAGs encoding homologs of cytochrome *c* oxidase (Cox I and II; EC 7.1.1.9), which is used to respire O_2_ in most aerobic or facultatively anaerobic bacteria and archaea (74). MAGs that did not encode Cox homologs (comprising all subtypes) were deemed anaerobes unless those MAGs were too incomplete to make a definitive determination. In such cases, literature related to the most closely affiliated strains was used to increase confidence in whether the organisms in question were likely to be capable of aerobic respiration or not. Finally, MAGs were examined for homologs of the cytochrome *bd* complex (CydABX; EC 7.1.1.7). In many organisms, CydABX is used to reduce O_2_ as a detoxification strategy (75), while in other organisms (currently only bacteria), it can be used to respire O_2_ (76). Thus, MAGs that encoded CydABX were conservatively labeled as aerotolerant unless physiological or biochemical evidence indicated that this complex in a closely related taxon is used to respire O_2_. An example is in the *Aquificae*, where aerobic respiration is catalyzed by CydABX (77, 78).

MAGs were also subjected to BLASTp analyses to identify homologs of proteins that may allow use of alternative electron donor and acceptor pairs that could potentially provide further insight into their ecology, including proteins involved in dissimilatory nitrate reduction (NarABG, EC 1.7.5.1, and NapAB, EC 1.9.6.1), sulfate/sulfite reduction (Sat, EC 2.7.7.4; AprAB, EC 1.8.99.2; DsrAB, EC 1.8.99.5), elemental sulfur/polysulfide reduction (DMSO reductases, EC 1.8.5.3; SreABC, no EC), sulfite/tetrathionate reduction (Asr, no EC), thiosulfate reduction (PhsA, EC 1.8.5.5), arsenate reduction (ArrA, EC 1.20.99.1), hydrogen metabolism ([NiFe] and [FeFe] hydrogenases, EC 1.12.1.2 and 1.12.99.6, respectively), arsenite oxidation (AioA, EC1.20.9.1), sulfide oxidation (Sqo, EC 1.8.5.8), thiosulfate/sulfur oxidation (Sox, EC 2.8.5.2), and methane oxidation or production (MmoX, EC 1.14.13.25; McrA, EC 2.8.4.1). A complete list of key enzymes and their EC numbers that were used to screen MAGs is provided in Table S3.

MAGs were also screened for pathways that allow for autotrophy. The six major autotrophic pathways are (i) the Calvin-Benson-Bassham (CBB) cycle, (ii) the reductive tricarboxylic acid (rTCA) cycle, (iii) the Wood-Ljungdahl (WL) pathway, (iv) the 3-hydroxypropionate (3HP) bicycle, (v) the 3-hydroxypropionate/4-hydroxybutyrate (3HP/4HB) cycle, and (vi) the dicarboxylate/4-hydroxybutyrate (DC/4HB) cycle (46, 47). Diagnostic proteins for each of these pathways were used to designate MAGs as being capable of autotrophy. This includes homologs of ribulose 1,5-bisphosphate carboxylase/oxygenase (RuBisCO; EC 4.1.1.39) and phosphoribulokinase (PRK; EC 2.7.1.19) for the CBB cycle and citryl-CoA synthetase (*ccs*; EC 6.2.1.18), citryl-CoA lyase (*cit*; EC 4.1.3.34), or ATP-citrate lyase (ACLY; EC 2.3.3.8) for the rTCA cycle. Homologs of carbon monoxide dehydrogenase/acetyl-CoA synthase (CODH/ACS; EC 1.2.7.4) and formate-tetrahydrofolate ligase (*fhs*; 6.3.4.3) were used to identify evidence for the Wood-Ljungdahl pathway, while homologs of propionyl-CoA carboxylase (PCC; EC 6.4.1.3), malonyl-CoA reductase (*mcr*; EC 1.2.1.75), malyl-CoA/B-methylmalyl-CoA/citranyl-lyase (*mcl*; EC 4.1.3.24), and acetyl-CoA carboxylase (ACC; EC 6.4.1.2) were used to identify evidence for the 3HP pathway. Evidence for the 3HP/4HB pathway was gained by identifying homologs of the same enzymes as the 3HP pathway with the addition of 4-hydroxybutyrl-CoA dehydratase (4-BUDH; EC 4.2.1.120). No enzyme homolog is diagnostic for the DC/4HB since it uses enzymes common to the rTCA or 3HP/4B cycles above. However, the DC/4HB pathway can be differentiated from the 3HP/4HB pathway by identifying homologs of pyruvate synthase (*por*; EC 1.2. 7.1) (79, 80). MAGs lacking homologs of these indicator proteins for autotrophic pathways were assigned as putatively heterotrophic unless otherwise noted.

### Genome replication rate.

The GRiD (Growth Rate Index) program (81) was used to estimate genome replication rates of MAGs in the GGS063 planktonic and sediment communities. GRiD estimates *in situ* genome replication rate by calculating genomic peak-to-trough read ratios by mapping read coverage at the inferred origin and terminus of the genome. This calculation assumes bidirectional replication of a circular genome proceeding from the origin. Quality control is assessed by read mapping to determine coverage levels of the DNA replication initiator protein-encoding gene, *dnaA*, and the replication termination site gene, *dif*. *dnaA* is typically localized near the origin (*ori*) of replication, *oriC* (82), and *dif* is typically localized near the terminus (*ter*) (83). The GRiD score is assumed to be accurate when *dnaA*/*oriC* and *ter*/*dif* read coverage ratios approach 1.0 (81). GRiD also estimates strain heterogeneity by determining variance in GRiD estimates across a single MAG. GRiD scores are assumed to be accurate when strain heterogeneity is less than 0.3 (81).

Replication rate estimates for all MAGs were performed using the GRiD single option, which estimates a replication rate for the MAG independent of the replication rates of the other community members. GRiD results of MAGs with *dif*/*ter* ratios outside the range 0.7 to 1.3, or species heterogeneity exceeding 0.3, were discarded without further consideration, as they did not meet quality thresholds. *dnaA*/*ori* ratios were not evaluated, as *dnaA* does not colocalize near the origin in most archaea (84), making it an uninformative metric for archaeal MAGs. It is important to note that some of the archaeal community members are closely related to organisms that are polyploid, such as *Archaeoglobus* (85). However, while many archaea are polyploid or may change ploidy over the course of their life cycle (86), the peak/trough ratio would be expected to be the same regardless of ploidy. Similarly, some archaeal community members are closely related to organisms encoding multiple origins of replication, such as *Pyrobaculum* (87). However, the empirical determination of low strain heterogeneity estimates for all archaeal MAGs that are related to organisms that putatively encode multiple origins of replications indicates that those MAGs likely encode only a single origin of replication.

GRiD score comparisons were initially generated for eight MAG pairs present in both the sediment and planktonic communities, as determined by pairwise average nucleotide identity (ANI) and amino acid identity (AAI) that each exceeded 95%. Pairwise ANI and AAI values for each MAG pair are presented in Table S4. Ninety-five percent was chosen as the cutoff because it consistently corresponds to the species level (88) and was presumed to minimize the confounding effects of differing phylogeny on growth rate (52). Pairs were only selected that met a minimum pairwise genome alignment of 20% to ensure selected pairs best represented the closest phylogenetic relatives. Consequently, some pairs, such as planktonic bin eight and sediment bin 36, were paired despite not sharing the highest pairwise ANI or AAI score. ANI scores were generated by FastANI (89). AAI scores were generated by using the AAI comparison workflow (aai_wf module) within the CompareM v.0.0.23 software package as described above. GRiD scores were then calculated for all MAGs with completeness >50%.

### Data availability.

Individual MAGs (Table S2) from the planktonic and sediment communities have been deposited in the National Center for Biotechnological Information database under BioProject accession numbers PRJNA568221 and PRJNA568220, respectively.

## References

[1] Hurwitz S, Lowenstern JB. 2014. Dynamics of the Yellowstone hydrothermal system. Rev Geophys 52:375–411. 10.1002/2014RG000452.

[2] Fournier RO. 1989. Geochemistry and dynamics of the Yellowstone National Park hydrothermal system. Annu Rev Earth Planet Sci 17:13–53. 10.1146/annurev.ea.17.050189.000305.

[3] Shock EL, Holland M, Meyer-Dombard DA, Amend JP, Osburn GR, Fischer TP. 2010. Quantifying inorganic sources of geochemical energy in hydrothermal ecosystems, Yellowstone National Park, USA. Geochim Cosmochim Acta 74:4005–4043. 10.1016/j.gca.2009.08.036.

[4] Inskeep W, Jay Z, Tringe S, Herrgard M, Rusch D, YNP Metagenome Project Steering Committee and Working Group Members. 2013. The YNP metagenome project: environmental parameters responsible for microbial distribution in the Yellowstone geothermal ecosystem. Front Microbiol 4:67. 10.3389/fmicb.2013.00067. 23653623 PMC3644721

[5] Colman DR, Lindsay MR, Amenabar MJ, Boyd ES. 2019. The intersection of geology, geochemistry, and microbiology in continental hydrothermal systems. Astrobiology 19:1505–1522. 10.1089/ast.2018.2016. 31592688

[6] Cox A, Shock EL, Havig JR. 2011. The transition to microbial photosynthesis in hot spring ecosystems. Chem Geol 280:344–351. 10.1016/j.chemgeo.2010.11.022.

[7] Boyd ES, Fecteau KM, Havig JR, Shock EL, Peters JW. 2012. Modeling the habitat range of phototrophs in Yellowstone National Park: toward the development of a comprehensive fitness landscape. Front Microbiol 3:221. 10.3389/fmicb.2012.00221. 22719737 PMC3376417

[8] Amend JP, Shock EL. 2001. Energetics of overall metabolic reactions of thermophilic and hyperthermophilic Archaea and Bacteria. FEMS Microbiol Rev 25:175–243. 10.1111/j.1574-6976.2001.tb00576.x. 11250035

[9] Amenabar MJ, Urschel MR, Boyd ES. 2015. Metabolic and taxonomic diversification in continental magmatic hydrothermal systems, p 57–96. In Bakermans C (ed), Microbial evolution under extreme conditions. De Gruyter, Berlin, Germany. 10.1515/9783110340716-006.

[10] Reysenbach A-L, Banta A, Civello S, Daly J, Mitchel K, Lalonde S, Konhauser K, Rodman A, Rusterholtz K, Takacs-Vesbach C, Inskeep WP, McDermott TR (ed). 2005. Aquificales in Yellowstone National Park. Thermal Biology Institute, Bozeman, MT.

[11] Takacs-Vesbach C, Inskeep WP, Jay ZJ, Herrgard MJ, Rusch DB, Tringe SG, Kozubal MA, Hamamura N, Macur RE, Fouke BW, Reysenbach AL, McDermott TR, Jennings R, Hengartner NW, Xie G. 2013. Metagenome sequence analysis of filamentous microbial communities obtained from geochemically distinct geothermal channels reveals specialization of three Aquificales lineages. Front Microbiol 4:84. 10.3389/fmicb.2013.00084. 23755042 PMC3665934

[12] Huber R, Eder W, Heldwein S, Wanner G, Huber H, Rachel R, Stetter KO. 1998. *Thermocrinis ruber* gen. nov., sp. nov., a pink-filament-forming hyperthermophilic bacterium isolated from Yellowstone National Park. Appl Environ Microbiol 64:3576–3583. 10.1128/AEM.64.10.3576-3583.1998. 9758770 PMC106467

[13] Colman DR, Feyhl-Buska J, Robinson KJ, Fecteau KM, Xu H, Shock EL, Boyd ES. 2016. Ecological differentiation in planktonic and sediment-associated chemotrophic microbial populations in Yellowstone hot springs. FEMS Microbiol Ecol 92:fiw137. 10.1093/femsec/fiw137. 27306555

[14] Sako Y, Nunoura T, Uchida A. 2001. *Pyrobaculum oguniense* sp. nov., a novel facultatively aerobic and hyperthermophilic archaeon growing at up to 97 degrees C. Int J Syst Evol Microbiol 51:303–309. 10.1099/00207713-51-2-303. 11321074

[15] Amo T, Paje MLF, Inagaki A, Ezaki S, Atomi H, Imanaka T. 2002. *Pyrobaculum calidifontis* sp. nov., a novel hyperthermophilic archaeon that grows in atmospheric air. Archaea 1:113–121. 10.1155/2002/616075. 15803649 PMC2685560

[16] Völkl P, Huber R, Drobner E, Rachel R, Burggraf S, Trincone A, Stetter KO. 1993. *Pyrobaculum aerophilum* sp. nov., a novel nitrate-reducing hyperthermophilic archaeum. Appl Environ Microbiol 59:2918–2926. 10.1128/aem.59.9.2918-2926.1993. 7692819 PMC182387

[17] Kashyap S, Sklute EC, Dyar MD, Holden JF. 2018. Reduction and morphological transformation of synthetic nanophase iron oxide minerals by hyperthermophilic archaea. Front Microbiol 9:1550. 10.3389/fmicb.2018.01550. 30050524 PMC6050373

[18] Jay ZJ, Beam JP, Dohnalkova A, Lohmayer R, Bodle B, Planer-Friedrich B, Romine M, Inskeep WP. 2015. *Pyrobaculum yellowstonensis* strain WP30 respires on elemental sulfur and/or arsenate in circumneutral sulfidic geothermal sediments of Yellowstone National Park. Appl Environ Microbiol 81:5907–5916. 10.1128/AEM.01095-15. 26092468 PMC4551270

[19] Stauffer RE, Thompson JM. 1984. Arsenic and antimony in geothermal waters of Yellowstone National Park, Wyoming, USA. Geochim Cosmochim Acta 48:2547–2561. 10.1016/0016-7037(84)90305-3.

[20] Cole JK, Peacock JP, Dodsworth JA, Williams AJ, Thompson DB, Dong H, Wu G, Hedlund BP. 2013. Sediment microbial communities in Great Boiling Spring are controlled by temperature and distinct from water communities. ISME J 7:718–729. 10.1038/ismej.2012.157. 23235293 PMC3605714

[21] Wang S, Dong H, Hou W, Jiang H, Huang Q, Briggs BR, Huang L. 2014. Greater temporal changes of sediment microbial community than its waterborne counterpart in Tengchong hot springs, Yunnan Province. Sci Rep 4:7479. 10.1038/srep07479. 25524763 PMC5378992

[22] He Q, Wang S, Hou W, Feng K, Li F, Hai W, Zhang Y, Sun Y, Deng Y. 2021. Temperature and microbial interactions drive the deterministic assembly processes in sediments of hot springs. Sci Tot Environ 772:145465. 10.1016/j.scitotenv.2021.145465. 33571767

[23] Gibson ML, Hinman NW. 2013. Mixing of hydrothermal water and groundwater near hot springs, Yellowstone National Park (USA): hydrology and geochemistry. Hydrogeol J 21:919–933. 10.1007/s10040-013-0965-4.

[24] Vitale MV, Gardner P, Hinman NW. 2008. Surface water–groundwater interaction and chemistry in a mineral-armored hydrothermal outflow channel, Yellowstone National Park, USA. Hydrogeol J 16:1381–1393. 10.1007/s10040-008-0344-8.

[25] Nye JJ, Shock EL, Hartnett HE. 2020. A novel PARAFAC model for continental hot springs reveals unique dissolved organic carbon compositions. Org Geochem 141:103964. 10.1016/j.orggeochem.2019.103964.

[26] Pearson FJ, Jr., Truesdell AH. 1978. Tritium in the waters of Yellowstone National Park, p 327–329. *In* Short Papers of the Fourth International Conference, Geochronology, Cosmochronology, Isotope Biology, 1978, U.S. Department of the Interior, Aspen, CO.

[27] Hurwitz S, Hunt AG, Evans WC. 2012. Temporal variations of geyser water chemistry in the Upper Geyser Basin, Yellowstone National Park, USA. Geochem Geophys 13:Q12005. 10.1029/2012GC004388.

[28] Gardner WP, Susong DD, Solomon DK, Heasler HP. 2011. A multitracer approach for characterizing interactions between shallow groundwater and the hydrothermal system in the Norris Geyser Basin area, Yellowstone National Park. Geochem Geophys 12:Q08005. 10.1029/2010GC003353.

[29] Nordstrom DK, McCleskey RB, Ball JW. 2009. Sulfur geochemistry of hydrothermal waters in Yellowstone National Park: IV acid-sulfate waters. Appl Geochem 24:191–207. 10.1016/j.apgeochem.2008.11.019.

[30] Bedinger MS, Pearson FJ, Jr., Reed JE, Sniegocki RT, Stone CG. 1979. The waters of Hot Springs National Park, Arkansas - their nature and origin. Geological Survey Professional Paper 1044-C. U.S. Government Printing Office, Washington, DC. 10.3133/pp1044C.

[31] Geng M, Duan Z. 2010. Prediction of oxygen solubility in pure water and brines up to high temperatures and pressures. Geochim Cosmochim Acta 74:5631–5640. 10.1016/j.gca.2010.06.034.

[32] McCleskey RB, Ball JW, Nordstrom DK, Holloway JM, Taylor HE. 2005. Water-chemistry data for selected springs, geysers, and streams in Yellowstone National Park Wyoming, 2001–2002. U.S. Geological Survey Open File Report 2004–1316. U.S. Department of the Interior, U.S. Geological Survey, Washington, DC. 10.3133/ofr20041316.

[33] Ballantyne JM, Moore JN. 1988. Arsenic geochemistry in geothermal systems. Geochim Cosmochim Acta 52:475–483. 10.1016/0016-7037(88)90102-0.

[34] Nordstrom DK, Ball JW, and McCleskey RB, Inskeep WP, McDermott TM (ed). 2005. Ground water to surface water: chemistry of thermal outflows in Yellowstone National Park. Thermal Biology Institute, Bozeman, MT.

[35] Amenabar MJ, Boyd ES. 2018. Mechanisms of mineral substrate acquisition in a thermoacidophile. Appl Environ Microbiol 84:e00334-18. 10.1128/AEM.00334-18. 29625980 PMC5981063

[36] Caporaso JG, Lauber CL, Walters WA, Berg-Lyons D, Lozupone CA, Turnbaugh PJ, Fierer N, Knight R. 2011. Global patterns of 16S rRNA diversity at a depth of millions of sequences per sample. Proc Nat Acad Sci USA 108:4516–4522. 10.1073/pnas.1000080107. 20534432 PMC3063599

[37] Walters W, Hyde ER, Berg-Lyons D, Ackermann G, Humphrey G, Parada A, Gilbert JA, Jansson JK, Caporaso JG, Fuhrman JA, Apprill A, Knight R. 2016. Improved bacterial 16S rRNA gene (V4 and V4-5) and fungal internal transcribed spacer marker gene primers for microbial community surveys. mSystems 1:e00009-15. 10.1128/mSystems.00009-15. PMC5069754 27822518

[38] Colman DR, Lindsay MR, Harnish A, Bilbrey EM, Amenabar MJ, Selensky MJ, Fecteau KM, Debes RV, Stott MB, Shock EL, Boyd ES. 2021. Seasonal hydrologic and geologic forcing drive hot spring geochemistry and microbial biodiversity. Environ Microbiol 23:4034–4053. 10.1111/1462-2920.15617. 34111905

[39] Meyer-Dombard DAR, Swingley W, Raymond J, Havig J, Shock EL, Summons RE. 2011. Hydrothermal ecotones and streamer biofilm communities in the Lower Geyser Basin, Yellowstone National Park. Environ Microbiol 13:2216–2231. 10.1111/j.1462-2920.2011.02476.x. 21453405

[40] Meyer-Dombard DR, Shock EL, Amend JP. 2005. Archaeal and bacterial communities in geochemically diverse hot springs of Yellowstone National Park, USA. Geobiology 3:211–227. 10.1111/j.1472-4669.2005.00052.x.

[41] Webb CO. 2000. Exploring the phylogenetic structure of ecological communities: an example for rain forest trees. Am Nat 156:145–155. 10.1086/303378. 10856198

[42] Schubotz F, Hays LE, Meyer-Dombard DAR, Gillespie A, Shock EL, Summons RE. 2015. Stable isotope labeling confirms mixotrophic nature of streamer biofilm communities at alkaline hot springs. Front Microbiol 6:42. 10.3389/fmicb.2015.00042. 25699032 PMC4318418

[43] Evans PN, Boyd JA, Leu AO, Woodcroft BJ, Parks DH, Hugenholtz P, Tyson GW. 2019. An evolving view of methane metabolism in the Archaea. Nat Rev Microbiol 17:219–232. 10.1038/s41579-018-0136-7. 30664670

[44] Gonsior M, Hertkorn N, Hinman N, Dvorski SEM, Harir M, Cooper WJ, Schmitt-Kopplin P. 2018. Yellowstone hot springs are organic chemodiversity hot spots. Sci Rep 8:14155. 10.1038/s41598-018-32593-x. 30237444 PMC6147864

[45] Schubotz F, Meyer-Dombard DR, Bradley AS, Fredricks HF, Hinrichs KU, Shock EL, Summons RE. 2013. Spatial and temporal variability of biomarkers and microbial diversity reveal metabolic and community flexibility in streamer biofilm communities in the Lower Geyser Basin, Yellowstone National Park. Geobiology 11:549–569. 10.1111/gbi.12051. 23981055

[46] Berg IA, Kockelkorn D, Ramos-Vera WH, Say RF, Zarzycki J, Hügler M, Alber BE, Fuchs G. 2010. Autotrophic carbon fixation in archaea. Nat Rev Microbiol 8:447–460. 10.1038/nrmicro2365. 20453874

[47] Berg IA. 2011. Ecological aspects of the distribution of different autotrophic CO_2_ fixation pathways. Appl Environ Microbiol 77:1925–1936. 10.1128/AEM.02473-10. 21216907 PMC3067309

[48] Kashyap S, Holden JF. 2021. Microbe-mineral interaction and novel proteins for iron oxide mineral reduction in the hyperthermophilic crenarchaeon *Pyrodictium delaneyi*. Appl Environ Microbiol 87:e02330-20. 10.1128/AEM.02330-20. 33419739 PMC8105010

[49] Amenabar MJ, Colman DR, Poudel S, Roden EE, Boyd ES. 2018. Electron acceptor availability alters carbon and energy metabolism in a thermoacidophile. Environ Microbiol 20:2523–2537. 10.1111/1462-2920.14270. 29749696

[50] Huber H, Stetter KO. 2015. Desulfurococcaceae. *In* Trujillo ME, Dedysh S, DeVos P, Hedlund B, Kämpfer P, Rainey FA, Whitman WB (ed), Bergey's manual of systematics of archaea and bacteria. John Wiley & Sons, Hoboken, NJ. 10.1002/9781118960608.fbm00085.

[51] Nakagawa S, Takai K, Horikoshi K, Sako Y. 2004. *Aeropyrum camini* sp. nov., a strictly aerobic, hyperthermophilic archaeon from a deep-sea hydrothermal vent chimney. Int J Syst Evol Microbiol 54:329–335. 10.1099/ijs.0.02826-0. 15023940

[52] Fagan WF, Pearson YE, Larsen EA, Lynch HJ, Turner JB, Staver H, Noble AE, Bewick S, Goldberg EE. 2013. Phylogenetic prediction of the maximum per capita rate of population growth. Proc R Soc B 280:2013.0523. 10.1098/rspb.2013.0523. PMC3774224 23720545

[53] Djokic T, Van Kranendonk MJ, Campbell KA, Walter MR, Ward CR. 2017. Earliest signs of life on land preserved in ca. 3.5 Ga hot spring deposits. Nat Comm 8:15263. 10.1038/ncomms15263. PMC5436104 28486437

[54] Damer B, Deamer D. 2020. The hot spring hypothesis for an origin of life. Astrobiology 20:429–452. 10.1089/ast.2019.2045. 31841362 PMC7133448

[55] Lyons TW, Reinhard CT, Planavsky NJ. 2014. The rise of oxygen in Earth’s early ocean and atmosphere. Nature 506:307–315. 10.1038/nature13068. 24553238

[56] St Clair B, Pottenger J, Debes R, Hanselmann K, Shock E. 2019. Distinguishing biotic and abiotic iron oxidation at low temperatures. ACS Earth Space Chem 3:905–921. 10.1021/acsearthspacechem.9b00016.

[57] Havig JR, Raymond J, Meyer-Dombard DAR, Zolotova N, Shock EL. 2011. Merging isotopes and community genomics in a siliceous sinter-depositing hot spring. J Geophys Res Biogeosci 116:G01005. 10.1029/2010JG001415.

[58] St-Jean G. 2003. Automated quantitative and isotopic (^13^C) analysis of dissolved inorganic carbon and dissolved organic carbon in continuous-flow using a total organic carbon analyser. Rapid Commun Mass Spectrom 17:419–428. 10.1002/rcm.926. 12590390

[59] Clum A, Huntemann M, Bushnell B, Foster B, Foster B, Roux S, Hajek PP, Varghese N, Mukherjee S, Reddy TBK, Daum C, Yoshinaga Y, O’Malley R, Seshadri R, Kyrpides NC, Eloe-Fadrosh EA, Chen I-MA, Copeland A, Ivanova NN. 2020. DOE JGI metagenome workflow. mSystems 6:e00804-20. 10.1128/mSystems.00804-20. PMC8269246 34006627

[60] Uritskiy GV, DiRuggiero J, Taylor J. 2018. MetaWRAP-a flexible pipeline for genome-resolved metagenomic data analysis. Microbiome 6:158. 10.1186/s40168-018-0541-1. 30219103 PMC6138922

[61] Li H, Durbin R. 2009. Fast and accurate short read alignment with Burrows-Wheeler transform. Bioinformatics 25:1754–1760. 10.1093/bioinformatics/btp324. 19451168 PMC2705234

[62] Kang DD, Li F, Kirton E, Thomas A, Egan R, An H, Wang Z. 2019. MetaBAT 2: an adaptive binning algorithm for robust and efficient genome reconstruction from metagenome assemblies. PeerJ 7:e7359. 10.7717/peerj.7359. 31388474 PMC6662567

[63] Wu YW, Simmons BA, Singer SW. 2016. MaxBin 2.0: an automated binning algorithm to recover genomes from multiple metagenomic datasets. Bioinformatics 32:605–607. 10.1093/bioinformatics/btv638. 26515820

[64] Alneberg J, Bjarnason BS, de Bruijn I, Schirmer M, Quick J, Ijaz UZ, Lahti L, Loman NJ, Andersson AF, Quince C. 2014. Binning metagenomic contigs by coverage and composition. Nat Methods 11:1144–1146. 10.1038/nmeth.3103. 25218180

[65] Parks DH, Imelfort M, Skennerton CT, Hugenholtz P, Tyson GW. 2015. CheckM: assessing the quality of microbial genomes recovered from isolates, single cells, and metagenomes. Genome Res 25:1043–1055. 10.1101/gr.186072.114. 25977477 PMC4484387

[66] Bowers RM, Kyrpides NC, Stepanauskas R, Harmon-Smith M, Doud D, Reddy TBK, Schulz F, Jarett J, Rivers AR, Eloe-Fadrosh EA, Tringe SG, Ivanova NN, Copeland A, Clum A, Becraft ED, Malmstrom RR, Birren B, Podar M, Bork P, Weinstock GM, Garrity GM, Dodsworth JA, Yooseph S, Sutton G, Glockner FO, Gilbert JA, Nelson WC, Hallam SJ, Jungbluth SP, Ettema TJG, Tighe S, Konstantinidis KT, Liu WT, Baker BJ, Rattei T, Eisen JA, Hedlund B, McMahon KD, Fierer N, Knight R, Finn R, Cochrane G, Karsch-Mizrachi I, Tyson GW, Rinke C, Lapidus A, Meyer F, Yilmaz P, Parks DH, Eren AM, et al. 2017. Minimum information about a single amplified genome (MISAG) and a metagenome-assembled genome (MIMAG) of bacteria and archaea. Nat Biotechnol 35:725–731. 10.1038/nbt.3893. 28787424 PMC6436528

[67] Chaumeil PA, Mussig AJ, Hugenholtz P, Parks DH. 2019. GTDB-Tk: a toolkit to classify genomes with the Genome Taxonomy Database. Bioinformatics 36:1925–1927. 10.1093/bioinformatics/btz848. PMC7703759 31730192

[68] Shannon P, Markiel A, Ozier O, Baliga NS, Wang JT, Ramage D, Amin N, Schwikowski B, Ideker T. 2003. Cytoscape: a software environment for integrated models of biomolecular interaction networks. Genome Res 13:2498–2504. 10.1101/gr.1239303. 14597658 PMC403769

[69] Kanehisa M, Goto S. 2000. KEGG: Kyoto Encyclopedia of Genes and Genomes. Nucleic Acids Res 28:27–30. 10.1093/nar/28.1.27. 10592173 PMC102409

[70] Moriya Y, Itoh M, Okuda S, Yoshizawa AC, Kanehisa M. 2007. KAAS: an automatic genome annotation and pathway reconstruction server. Nucleic Acids Res 35:W182–5. 10.1093/nar/gkm321. 17526522 PMC1933193

[71] Altschul SF, Gish W, Miller W, Myers EW, Lipman DJ. 1990. Basic local alignment search tool. J Mol Biol 215:403–410. 10.1016/S0022-2836(05)80360-2. 2231712

[72] Waterhouse A, Bertoni M, Bienert S, Studer G, Tauriello G, Gumienny R, Heer FT, de Beer TAP, Rempfer C, Bordoli L, Lepore R, Schwede T. 2018. SWISS-MODEL: homology modelling of protein structures and complexes. Nucleic Acids Res 46:W296–W303. 10.1093/nar/gky427. 29788355 PMC6030848

[73] Sievers F, Wilm A, Dineen D, Gibson TJ, Karplus K, Li W, Lopez R, McWilliam H, Remmert M, Söding J, Thompson JD, Higgins DG. 2011. Fast, scalable generation of high-quality protein multiple sequence alignments using Clustal Omega. Mol Syst Biol 7:539. 10.1038/msb.2011.75. 21988835 PMC3261699

[74] Ludwig B. 1987. Cytochrome *c* oxidase in prokaryotes. FEMS Microbiol Rev 3:41–56. 10.1111/j.1574-6968.1987.tb02451.x.

[75] Jünemann S. 1997. Cytochrome *bd* terminal oxidase. Biochim Biophys Acta 1321:107–127. 10.1016/s0005-2728(97)00046-7. 9332500

[76] Borisov VB, Gennis RB, Hemp J, Verkhovsky MI. 2011. The cytochrome *bd* respiratory oxygen reductases. Biochim Biophys Acta 1807:1398–1413. 10.1016/j.bbabio.2011.06.016. 21756872 PMC3171616

[77] Deckert G, Warren PV, Gaasterland T, Young WG, Lenox AL, Graham DE, Overbeek R, Snead MA, Keller M, Aujay M, Huber R, Feldman RA, Short JM, Olsen GJ, Swanson RV. 1998. The complete genome of the hyperthermophilic bacterium *Aquifex aeolicus*. Nature 392:353–358. 10.1038/32831. 9537320

[78] Schütz M, Brugna M, Lebrun E, Baymann F, Huber R, Stetter KO, Hauska G, Toci R, Lemesle-Meunier D, Tron P, Schmidt C, Nitschke W. 2000. Early evolution of cytochrome *bc* complexes. J Mol Biol 300:663–675. 10.1006/jmbi.2000.3915. 10891261

[79] Hügler M, Sievert SM. 2011. Beyond the Calvin cycle: autotrophic carbon fixation in the ocean. Annu Rev Mar Sci 3:261–289. 10.1146/annurev-marine-120709-142712. 21329206

[80] Hawkins AS, Han Y, Bennett RK, Adams MW, Kelly RM. 2013. Role of 4-hydroxybutyrate-CoA synthetase in the CO_2_ fixation cycle in thermoacidophilic archaea. J Biol Chem 288:4012–4022. 10.1074/jbc.M112.413195. 23258541 PMC3567653

[81] Emiola A, Oh J. 2018. High throughput in situ metagenomic measurement of bacterial replication at ultra-low sequencing coverage. Nat Comm 9:4956. 10.1038/s41467-018-07240-8. PMC6251912 30470746

[82] Mackiewicz P, Zakrzewska-Czerwinska J, Zawilak A, Dudek MR, Cebrat S. 2004. Where does bacterial replication start? Rules for predicting the *oriC* region. Nucleic Acids Res 32:3781–3791. 10.1093/nar/gkh699. 15258248 PMC506792

[83] Hendrickson H, Lawrence JG. 2007. Mutational bias suggests that replication termination occurs near the *dif* site, not at Ter sites. Mol Microbiol 64:42–56. 10.1111/j.1365-2958.2007.05596.x. 17376071

[84] Wu Z, Liu J, Yang H, Xiang H. 2014. DNA replication origins in archaea. Front Microbiol 5:179. 10.3389/fmicb.2014.00179. 24808892 PMC4010727

[85] Maisnier-Patin S, Malandrin L, Birkeland N-K, Bernander R. 2002. Chromosome replication patterns in the hyperthermophilic euryarchaea *Archaeoglobus fulgidus* and *Methanocaldococcus* (*Methanococcus*) *jannaschii*. Mol Microbiol 45:1443–1450. 10.1046/j.1365-2958.2002.03111.x. 12207709

[86] Breuert S, Allers T, Spohn G, Soppa J. 2006. Regulated polyploidy in halophilic Archaea. PLoS One 1:e92. 10.1371/journal.pone.0000092. 17183724 PMC1762399

[87] Pelve EA, Lindås A-C, Knöppel A, Mira A, Bernander R. 2012. Four chromosome replication origins in the archaeon *Pyrobaculum calidifontis*. Mol Microbiol 85:986–995. 10.1111/j.1365-2958.2012.08155.x. 22812406

[88] Konstantinidis KT, Ramette A, Tiedje JM. 2006. The bacterial species definition in the genomic era. Philos Trans R Soc Lond B Biol Sci 361:1929–1940. 10.1098/rstb.2006.1920. 17062412 PMC1764935

[89] Jain C, Rodriguez-R LM, Phillippy AM, Konstantinidis KT, Aluru S. 2018. High throughput ANI analysis of 90K prokaryotic genomes reveals clear species boundaries. Nat Comm 9:5114. 10.1038/s41467-018-07641-9. PMC6269478 30504855

[90] Ball JW, McCleskey RB, Nordstrom DK, Holloway JM. 2008. Water-chemistry data for selected springs, geysers, and streams in Yellowstone National Park, Wyoming, 2003–2005. U.S. Geological Survey Open File Report 2006–1339. U.S. Department of the Interior, U.S. Geological Survey, Washington, DC. 10.3133/ofr20061339.

