# Supplementary material for: Ecological Dichotomies Arise in Microbial Communities Due to Mixing of Deep Hydrothermal Waters and Atmospheric Gas in a Circumneutral Hot Spring
Source: Appl Environ Microbiol. 2021 Nov 10;87(23):e01598-21. doi: 10.1128/AEM.01598-21 (PMC8579995; doi:10.1128/AEM.01598-21)
Supplement: Supplemental file 1 — Fig. S1. Download aem.01598-21-s0001.pdf, PDF file, 0.2 MB [file aem.01598-21-s0001.pdf]

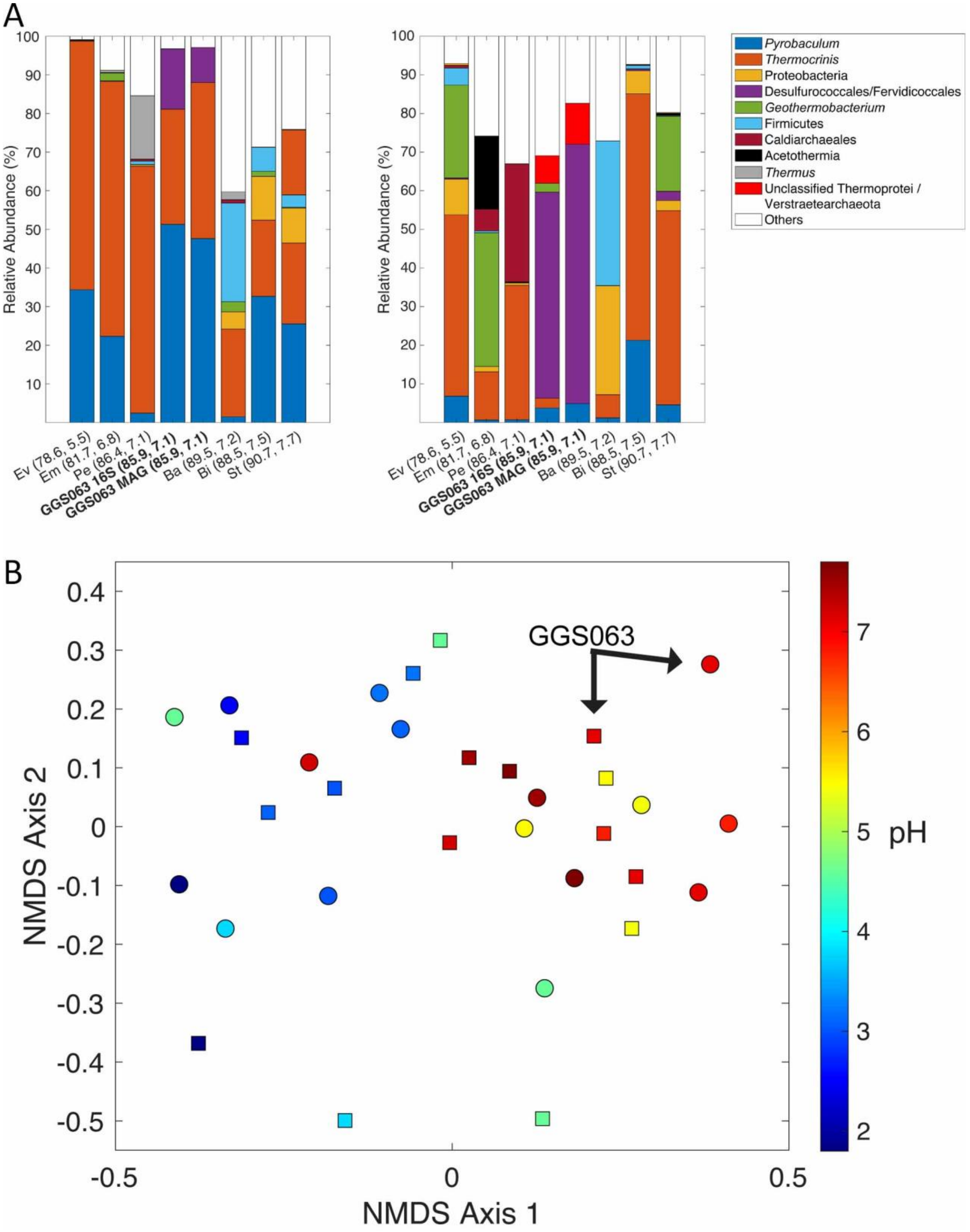

Supplemental Figure 1. (**A**) Bar charts comparing the taxonomy of metagenome assembled genomes (MAGs) recovered from planktonic (left) and sediment (right) communities from GGS063 (GGS063 MAG), 16S rRNA genes extracted from planktonic and sediment

metagenomic assemblies of GGS063 (GGS063 MAG), and 16S rRNA genes previously amplified and sequenced from planktonic and sediment communities collected from geochemically comparable springs in Yellowstone National Park, Wyoming, U.S.A. (1). The pH and temperatures of the springs where those samples were collected are indicated. (**B**) Non-metric multidimensional scaling ordination of 16S rRNA gene operational taxonomic unit (OTU) compositional profiles previously amplified and sequenced from DNA extracted from planktonic and sediment communities based on PCR amplification of 16S rRNA genes from 15 hot springs in Yellowstone National Park, Wyoming, U.S.A. (1) with the addition of 16S rRNA genes extracted from metagenomic assemblies of DNA extracted from GGS063. Points are colored by spring pH, given by the scale on the right. Squares indicate planktonic communities and circles indicate sediment communities. Additional taxonomic and geochemical information for the samples used in this comparative analysis can be found in Colman et al., 2016 (1).

References

1. Colman DR, Feyhl-Buska J, Robinson KJ, Fecteau KM, Xu H, Shock EL, and Boyd ES. 2016. Ecological differentiation in planktonic and sediment-associated chemotrophic microbial populations in Yellowstone hot springs. FEMS Microbiol Ecol 92:fiw137. https://doi.org/10.1093/femsec/fiw137.
